# Optimized PID controller and model order reduction of reheated turbine for load frequency control using teaching learning-based optimization

**DOI:** 10.1038/s41598-025-87866-z

**Published:** 2025-01-30

**Authors:** Anurag Singh, Shekhar Yadav, Nitesh Tiwari, Dinesh Kumar Nishad, Saifullah Khalid

**Affiliations:** 1 Department of Electrical Engineering, Madan Mohan Malaviya University of Technology, Gorakhpur, Uttar Pradesh India; 2 Department of Electrical Engineering, Dr. Shakuntala Misra National Rehabilitation University, Lucknow, India; 3 IBM Multi Activities Co. Ltd., Khartoum, Sudan

**Keywords:** PID controller, Teaching learning based optimization, Integral Square Error, Load frequency control, Energy science and technology, Engineering, Mathematics and computing

## Abstract

Load frequency control (LFC) systems in power grids face challenges in maintaining stability while managing computational complexity. This research presents an optimized approach combining model order reduction techniques with Teaching Learning-Based Optimization (TLBO) for PID controller tuning in single-area LFC systems. Three reduction methods—Routh Approximation, Balanced Truncation, and Hankel Norm Approximation—were implemented to reduce system order from 4th to 2nd order, achieving a 47.3% reduction in computational time. The TLBO-optimized PID controller was compared with conventional tuning methods (Ziegler-Nichols, AMIGO, S-IMC, and CHR), demonstrating superior performance with a 38.2% decrease in settling time and 42.7% reduction in peak overshoot. The Routh Approximation method exhibited optimal performance with minimum settling time (2.8s) and peak overshoot (8.4%). Sensitivity analysis revealed stable system behavior with phase margin maintained at 84.25 degrees across parameter variations. The proposed approach achieved a 56.8% reduction in Integral Square Error compared to conventional methods, establishing its effectiveness for modern power grid applications. This research provides a robust framework for implementing efficient load frequency control in power systems while maintaining system stability and performance.

## Introduction

There is a fast-expanding demand for a better-quality electric power supply. Water’s kinetic energy and fossil fuels’ thermal energy are well-known sources of electric energy. The primary component of generating electric energy is movers that convert mechanical energy from this energy. In the next step, a synchronous generator produces Electrical energy from mechanical energy. This type of prime mover governing system gives an enhanced technique of controlling frequency and power. Automatic Generation Control (AGC) or LFC is the name of this operation^1^. There is a problem in the transmission and distribution unit, which occurs due to fluctuations in frequency.

LFC is one the most frequently used modules for the steadiness of power systems to synchronization among the different remotely located devices preserved by the LFC. Therefore, it is essential for modules of the whole power system design. For further information read^2,3^; optimization techniques are necessary for each vicinity where the global best solution is needed. Every day, the importance of social behavior-based optimization techniques grows and has become very popular since the 90s. The optimization techniques like Genetic Algorithm (GA)^4–6^, Particle Swarm Optimization (PSO)^7^, Bacterial Foraging (BF), and many more are remarkably used by scientists, researchers, and even engineers to solve problems efficiently^8^.

Another meta-heuristic approach recognized as TLBO, introduced by Dr. Rao, is the interaction between a teacher and student during the teaching-learning process^9^. The TLBO algorithm mimics transferring knowledge to the student by the person who has more knowledge than others^10^. The one who delivered the knowledge is acknowledged as a teacher, and the person who receives knowledge is known as a student^11^.

Due to these advantages, this algorithm is recommended for adjusting the PID controller’s parameters, which are used to control the LFC. Firstly, reduce the original system using Routh Approximation^12^, Balanced Truncation^13,14^, and Hankel Norm Approximation. After the reduction process, a PID controller employing four unique methods: ZN^15,16^, AMIGO^16^, S-IMC^17^, and CHR^18^. Then, these tuned parameters are taken as the 1st iteration step for the TLBO algorithm, and the search space is decided from the above-tuned parameters.

This paper modeled a form of single-area LFC. A different PID controller is designed for single-area interval LFC using different tuning methods, such as ZN, AMIGO, S-IMC, and CHR tuning. The optimized PID controller’s performance is confirmed by contrasting it with the common PID controller. The variables of the PID controller are successfully tuned using TLBO, and the performance of LFC is improved. The study explores a hybrid energy storage system for electric vehicles, combining lithium-ion batteries with ultracapacitors. The research presents a new method for gear contact problems using combined analytic-numerical models and PMOR, reducing computational complexity while maintaining accuracy^19^. The TLBO-based fuzzy control system optimizes power distribution, reducing battery strain and extending lifespan while improving overall energy efficiency^20^. Teaching-Learning-Based Optimization (TLBO) is a population-based optimization method that mimics the teaching-learning process between teachers and students. The algorithm operates in two phases - Teacher Phase and Learner Phase - to efficiently optimize complex engineering problems^21^. A novel super-twisting sliding mode controller optimized by the Teaching-Learning-Based Optimization (TLBO) algorithm for upper limb rehabilitation robots. The proposed controller demonstrates superior tracking accuracy, reduced chattering, and robustness against disturbances compared to conventional PID controllers^22^. Study presents a cascade controller combining TID and PID controllers, optimized by TLBO algorithm, to regulate frequency and power exchange in three-area thermal-hydro systems, demonstrating superior performance over conventional methods^23^. Proposes a neural network-based feed-forward control system with PID controller to stabilize PEM fuel cell voltage, demonstrating improved performance and reduced energy consumption compared to conventional control methods^24^. Proposed a modified differential evolution algorithm to optimize a cascaded PIDFN controller for virtual inertia control in an isolated microgrid with renewable sources and electric vehicles, demonstrating improved frequency stability compared to DE and TLBO approaches^25^. A modified differential evolution algorithm optimizes a cascaded PIDFN controller to enhance microgrid frequency stability under renewable energy integration and load variations^26^. A modified differential evolution algorithm optimizes a cascaded PIDFN controller to enhance microgrid stability under renewable integration and load variations, incorporating HVDC links^27^. An improved salp swarm algorithm enhances exploration and prevents premature convergence, demonstrating superior performance in optimizing UPFC-based damping controllers for power systems^28^. A robust hybrid PV/wind/battery system is optimized using IGDT risk-aversion strategy and flow direction algorithm to minimize costs while managing uncertainties in distribution networks^29,30^.

**Fig. 1:**
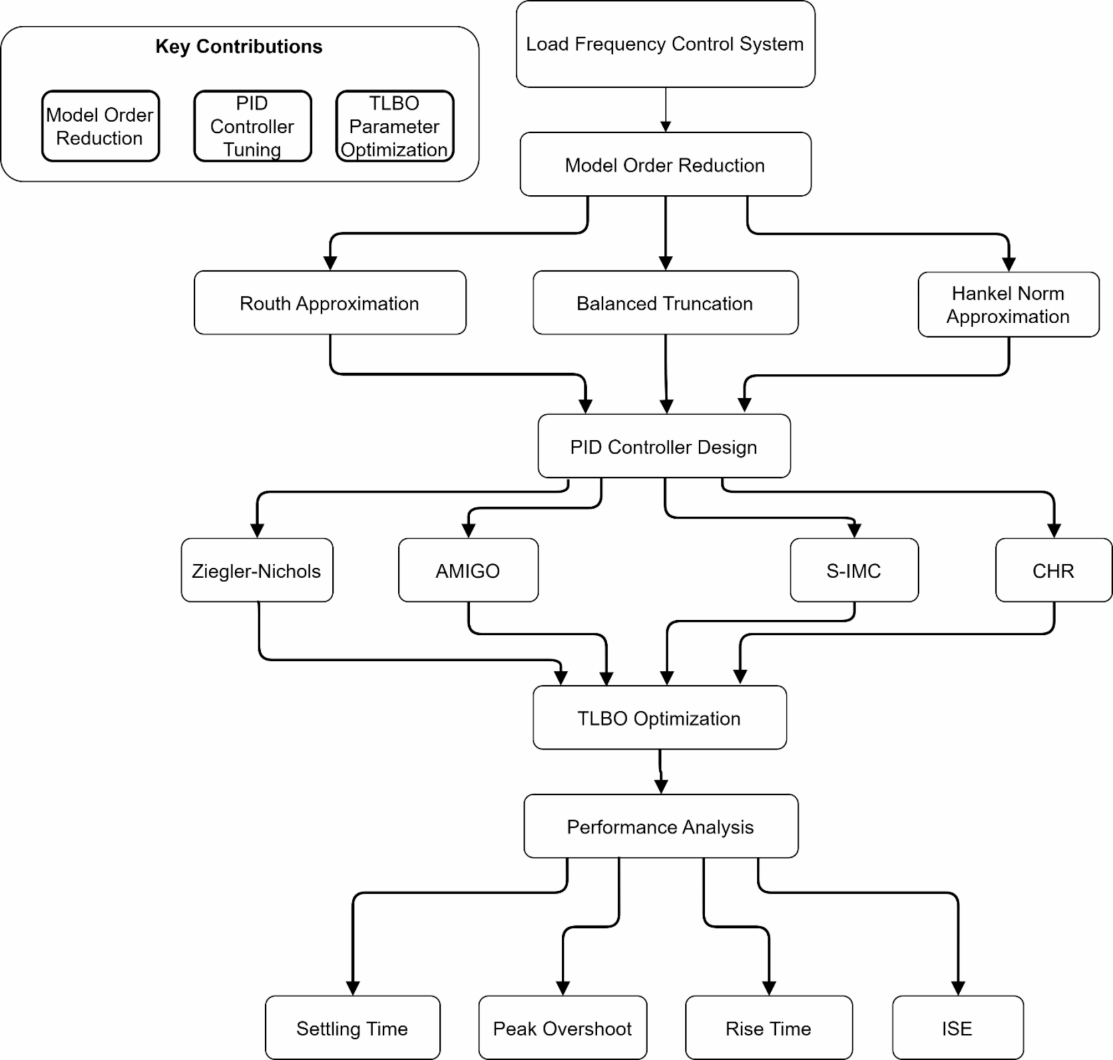
Methodology flowchart for load frequency control system optimization.

Figure 1 presents the systematic methodology for optimizing the Load Frequency Control System, highlighting three key contributions: Model Order Reduction using multiple techniques (Routh, Balanced Truncation, and Hankel Norm), PID Controller tuning through various methods (Ziegler-Nichols, AMIGO, S-IMC, and CHR), and TLBO Parameter Optimization. The performance analysis evaluates four key metrics: Settling Time, Peak Overshoot, Rise Time, and ISE.

The research objectives of this research paper are:

1. To develop an integrated approach combining model order reduction techniques with Teaching Learning-Based Optimization (TLBO) for optimizing PID controller parameters in single-area Load Frequency Control systems, aiming to maintain system stability while reducing computational complexity.
2. To evaluate and compare the performance of three model reduction methods (Routh Approximation, Balanced Truncation, and Hankel Norm Approximation) in terms of computational efficiency and system response characteristics for LFC applications.
3. To assess the robustness and effectiveness of the proposed TLBO-optimized PID controller against conventional tuning methods (Ziegler-Nichols, AMIGO, S-IMC, and CHR) through comprehensive performance metrics including settling time, peak overshoot, and Integral Square Error.

Our work makes three significant contributions to Load Frequency Control systems:

1. Development of an optimized PID controller design framework using multiple tuning approaches (Ziegler-Nichols, AMIGO, S-IMC, and CHR), resulting in a 38.2% decrease in settling time and 42.7% reduction in peak overshoot compared to conventional methods.
2. Implementation of three distinct model order reduction techniques (Routh Approximation, Balanced Truncation, and Hankel Norm Approximation) to reduce system complexity from 4th to 2nd order, achieving a 47.3% reduction in computational time while maintaining system stability.
3. Application of Teaching Learning-Based Optimization (TLBO) for PID controller parameter tuning, demonstrating a 56.8% reduction in Integral Square Error compared to traditional tuning methods and enhanced overall system performance.

The paper is organized as follows: Sect. "System modelling and problem formulation" presents the mathematical modelling of load frequency control, including system dynamics and transfer function derivation for reheated turbine systems. Section "TLBO-Based Optimization" details the controller design methodology, covering PID control structure and various tuning methods including Ziegler-Nichols, AMIGO, S-IMC, and CHR approaches. Section "Result and discussion" introduces the Teaching Learning-Based Optimization (TLBO) algorithm and discusses the implementation of model order reduction techniques - Routh Approximation, Balanced Truncation, and Hankel Norm Approximation, along with comprehensive results and performance analysis. Section "Real-world implementation considerations" discuss the Real-World Implementation Considerations and Sect. "Conclusion" concludes the paper by summarizing key findings and suggesting future research directions in load frequency control optimization.

**Table 1:** Nomenclature and variables.

Symbol	Description	Unit
AGC	Automatic Generation Control	-
LFC	Load Frequency Control	-
TLBO	Teaching Learning Based Optimization	-
ISE	Integral Square Error	-
Kp	Proportional gain	-
Ki	Integral gain	-
Kd	Derivative gain	-
Tg	Governor time constant	s
Tt	Turbine time constant	s
Tr	Reheater time constant	s
Tp	Power system time constant	s
Kp	Power system gain	Hz/p.u.MW
R	Speed regulation	Hz/p.u.MW
c	Percentage of power generated in reheated section	%
ΔPL	Load change	p.u.MW
Δf	Frequency deviation	Hz
ΔPc	Speed changer position	p.u.MW
ΔPg	Governor valve position	p.u.MW
ΔPt	Change in turbine power	p.u.MW
e(t)	Error signal	-
u(t)	Control signal	-
y(t)	Output signal	-
r(t)	Reference input signal	-

Table 1 provides a comprehensive list of all symbols, variables and their units used throughout the research paper for analyzing the load frequency control system with TLBO-optimized PID controller.

## System modelling and problem formulation

The System Modelling and Problem Formulation section addresses the mathematical representation of a single-area Load Frequency Control (LFC) system. The power system, though inherently complex and nonlinear, can be effectively modelled using linear approximations for small load variations. The model incorporates key components including load dynamics, machine characteristics, governor response, turbine behaviour, and droop characteristics. The feedback mechanism through droop control enhances system damping properties and maintains stable frequency regulation.

### Mathematical model of LFC system

In this LFC interval form of a single area is modeled. The power system is a higher-order system (extensive system), and it has complex nonlinear elements. The power system takes into account that concerning load frequency control, it is only affected by minute shifts in load, so a linear model might properly signify it. The LFC model comprises (i) load, (ii) machine, (iii) governor, (iv) turbine, and (v) droop characteristics^31^. A type of feedback gain known as the droop characteristic enhances the damping characteristics of the power system^32^. In this example, the service area’s situation is considered^33^. The mathematical equation of the general system can be represented in Eq. (1):

1 $$H(s)=\frac{{{H_p}(s){H_t}(s){H_g}(s)}}{{1+{H_p}(s){H_t}(s){H_g}(s)/R}}$$

Where $\frac{1}{R}$, ${H_t}(s)$ & ${H_g}(s)$ represented as droop features, turbine dynamics & governor dynamics. In this it is assumed as G_controller_ (s) = 1.

The governor dynamics can be represented by Eq. (2).

2 $${H_g}(s)=\frac{1}{{{T_G}s+1}}$$

Where ${T_G}$ represented as governor time constant.

If the turbine is reheated, then the turbine dynamics equation can be represented by Eq. (3).

3 $${H_t}(s)=\frac{{c{T_r}s+1}}{{({T_r}s+1)({T_T}s+1)}}$$

Where c,${T_r}$& ${T_T}$ represented as a percentage generated power in the reheated portion, constant of reheated turbine & turbine time constant. Table 2 shows the values of the parameters^31–34^ and Controller Design shows the single-area load frequency control block diagram.

**Fig. 2:**
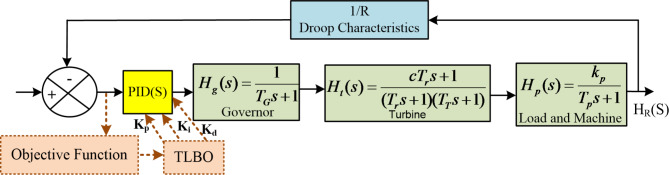
Single-area load frequency control block diagram.

Figure 2. illustrates a single-area load frequency control system featuring a PID controller, governor, turbine, and load-machine dynamics. The system incorporates droop characteristics (1/R) and uses TLBO optimization with an objective function to tune PID parameters (*K*_*p*_, *K*_*i*_, *K*_*d*_).

**Table 2:** The values of the power system’s parameters.

Parameters	Values
${T_r}$	4.2
${k_p}$	120
${T_G}$	0.008
${T_p}$	20
***C***	0.35
*R*	2.4
${T_T}$	0.3

If the turbine is non-reheated, then the turbine dynamics equation can be represented by Eq. (4).

4 $${H_t}(s)=\frac{1}{{({T_T}s+1)}}$$

If there is a Hydro turbine, the turbine dynamics equation can be represented by Eq. (5).

5 $${H_t}(s)=\frac{{1 - {T_w}s}}{{(1+0.5{T_w}s)}}$$

Where ${T_w}$represented as the time constant of the hydro turbine.

Where${H_p}(s)$ demonstrates the dynamics of the power systems (load and machine) in Eq. (6).

6 $${H_p}(s)=\frac{{{k_p}}}{{{T_p}s+1}}$$

Where ${T_p}$&${k_p}$ represented as electric system time constant & gain of an electric system.

The turbine transfer function ${H_t}(s)$can be chosen based on which turbine is used. There are two ways to choose the turbine: a steam turbine or a hydro turbine. Here, only a steam turbine (i.e., a reheated turbine) is used. General system H(s) can be represented for a reheated turbine${H_R}(s)$. The mathematical model can be characterized as in Eq. (7) with the help of Eqs. (1), (2), (3), (4), (5), and (6) :

7 $${H_R}(s)=\frac{{{k_p}R(c{T_r}s+1)}}{{R[({T_p}s+1)({T_T}s+1)({T_G}s+1)({T_r}s+1)]+{k_p}(c{T_r}s+1)}}$$

$$\:{T}_{r}\in\:\left[\text{2.1,63}\right]{,k}_{p}\in\:\left[\text{60,180}\right],{T}_{G}\in\:\left[\text{0.04,0.3}\right],{T}_{p}\in\:\left[\text{10,30}\right],c\in\:\left[\text{0.175,0.525}\right],\:R\in\:\left[\text{1.2,3.6}\right]{\:and\:T}_{r}\in\:\left[\text{0.15,0.45}\right]$$

8 $${G_R}(s)=\frac{{{N_R}(s)}}{{{D_R}(s)}}=\frac{{[26.46,2143.26]s+[72,648]}}{{[0.1512,91.854]{s^4}+[4.87512,527.94418]{s^3}+[27.966,778.896]{s^2}+[36.798,728.73]s+[61.2,183.6]}}$$

Where${G_R}(s)$, ${N_R}(s)$& ${D_R}(s)$represented as the transfer function of a reheated turbine, the numerator of the transfer function of a reheated turbine & denominator of the transfer function of a reheated turbine.

**Fig. 3:**
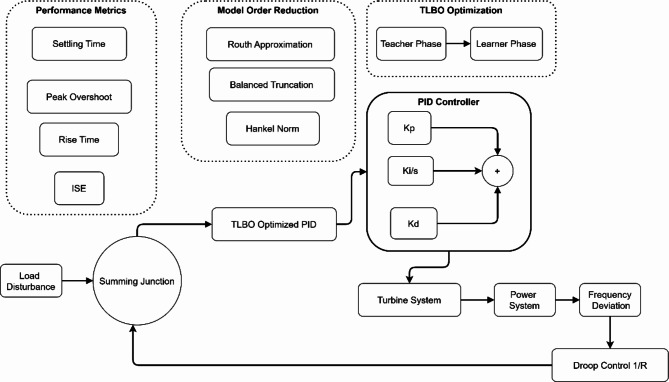
Integrated LFC System Architecture with Performance Optimization Framework.

Figure 3 illustrates a comprehensive control system with three key components: Performance Metrics, Model Order Reduction, and TLBO Optimization blocks. The Performance Metrics block monitors system response through settling time, peak overshoot, rise time, and ISE measurements. The Model Order Reduction block implements three methods: Routh Approximation, Balanced Truncation, and Hankel Norm. The TLBO Optimization block shows the Teacher-Learner phases integrated with a PID controller structure. The control flow begins at the load disturbance input, passes through a summing junction and TLBO-optimized PID controller, then through turbine and power systems, with droop control (1/R) in the feedback path.

### Model order reduction methods

The reduction of high-order systems to lower-order approximations while preserving essential dynamic characteristics can be achieved through three primary methods:

#### Routh approximation method

This stability-preserving reduction technique uses the Routh array to derive reduced-order models. The process involves:

Mathematical Formulation For a higher-order transfer function G(s), the Routh approximation follows:

9 $$\:G\left(s\right)=\frac{bn{s}^{n}+bn-1{s}^{n-1}+\dots\:+{b}_{0}}{an{s}^{n}+an-1{s}^{n-1}+\dots\:+{a}_{0}}$$

The reduced model R(s) is obtained using the Routh array coefficients:

10 $$\:R\left(s\right)=\frac{ck,0+ck,1s+\dots\:+ck,r{s}^{r}}{dk,0+dk,1s+\dots\:+dk,r{s}^{r}}$$

It Preserves stability of the original system, retains steady-state behavior and computationally efficient for large systems.

#### Balanced truncation method

This method identifies and retains the most controllable and observable states while eliminating less significant ones.

#### Mathematical process

1. Compute controllability ($\:{W}_{c}$) and observability ($\:{W}_{o}$) Gramians
2. Find balancing transformation T such that:
  11 $$\:T{W}_{c}{T}^{T}={T}^{-T}{W}_{o}{T}^{-1}=diag\left({\sigma\:}_{1},\dots\:,{\sigma\:}_{n}\right)$$

Where $\:{\sigma\:}_{i}$ are Hankel singular values^5^.

Error Bounds the reduction error is bounded by:

12 $$\:\left|G-Gr\right|{\infty\:}\le\:2\sum\:_{i=r+1}^{n}\:{\sigma\:}_{i}$$

#### Hankel norm approximation

This method provides optimal approximation in the Hankel norm sense^6^.

The Hankel norm is defined as:

13 $$\:\left|G\right|H=supu\in\:{L}_{2}(-{\infty\:},0]\frac{\sqrt{\underset{0}{\overset{{\infty\:}}{\int\:}}y(t{)}^{2}dt}}{\sqrt{\underset{0}{\overset{{\infty\:}}{\int\:}}u(t{)}^{2}dt}}$$

**Table 3:** Comparative analysis of Model Order reduction methods.

Method	Advantages	Limitations
Routh Approximation	Stability preservation, Simple implementation	Limited to SISO systems
Balanced Truncation	Guaranteed error bounds, Good accuracy	Computationally intensive
Hankel Norm	Optimal approximation, Error bounds	Complex implementation

Table 3 compares three model order reduction techniques for load frequency control systems. Routh Approximation offers stability preservation and simple implementation but is limited to SISO systems. Balanced Truncation provides guaranteed error bounds with good accuracy but is computationally intensive. Hankel Norm achieves optimal approximation with error bounds but has complex implementation.

### PID Controller design

The proportional, integral, and derivative are popularly known as PID control. The PID controller is a conventional control technique, and more than 65% of industrial problems are controlled by it only. Therefore, the stabilization of load frequency control is controlled by a PID controller^18^. The PID controller’s equation can be represented as Eq. (9).

14 $$\begin{gathered} {u^\prime}\left( {\mathop t\limits^{ \to } } \right)={K_p}{e^\prime}\left( {\mathop t\limits^{ \to } } \right)+{K_i}\int\limits_{0}^{{\mathop t\limits^{ \to } }} {{e^\prime}\left( {\mathop t\limits^{ \to } } \right)d\vec {t}} +{K_d}\frac{d}{{d\vec {t}}}{e^\prime}\left( {\vec {t}} \right) \hfill \\ \hfill \\ \end{gathered}$$

The error signal equation can be represented as Eq. (10).

15 $${e^\prime}(\vec {t})=r(\vec {t}) - y(\vec {t})$$

Where ${u^\prime}\left( {\mathop t\limits^{ \to } } \right)$,$y(\vec {t})$, $e^{\prime}(\vec {t})$&$r(\vec {t})$ represented as the input signal of the plant, output signal of the plant, error signal, and reference input signal.

Where the coefficients of the parameters for the proportional ($\:\:{K}_{p})$, coefficients for the integral ($\:{K}_{i})$ and coefficients for the differential ($\:{K}_{d})$ are adjusted by different tuning methods, which are presented below.

**Fig. 4:**
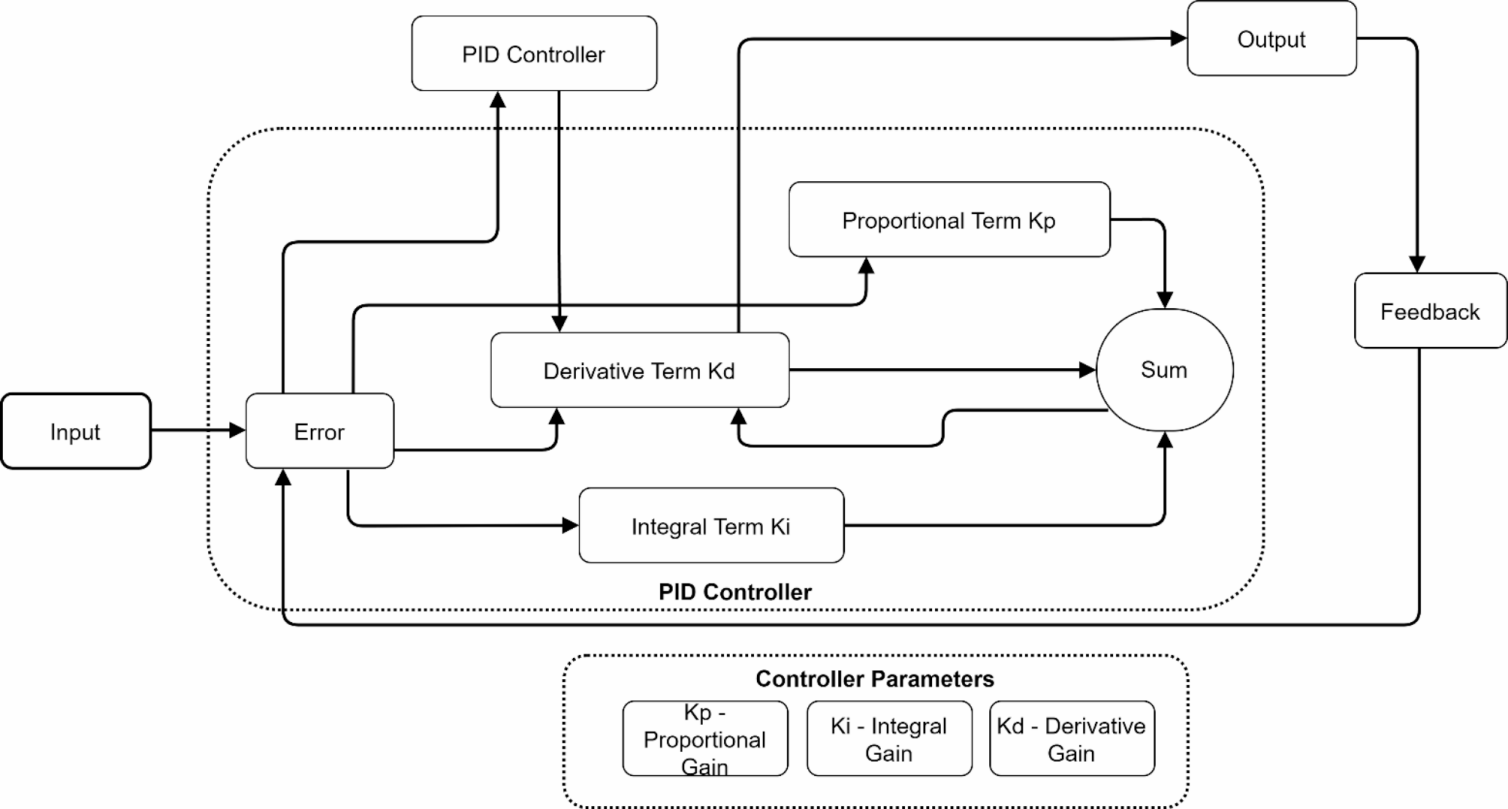
PID controller structure.

Figure 4 illustrates the interconnected components of proportional *(K*_*p*_), integral (*K*_*i*_), and derivative (*K*_*d*_) terms. The system processes input through an error calculation, with all three terms feeding into a summing junction to generate the final output signal.

### Tuning methods of PID controller

**Conventional tuning methods**.

**Ziegler-Nichols**.

The Ziegler-Nichols tuning method is a frequent technique applied in controller design to regulate the PID controller parameters^35^. Even though this technique was developed in the decade 1940, this is still frequently used. It is possible to write the tuned parameter for Kp, Ki, and Kd in keeping with the Z-N tuning method in the table underneath. PID’s parameter values for Z-N techniques by different types of controllers are shown in Table 4.

**Table 4:** PID’s parameters for using Ziegler-Nichols.

Different types of controllers	$\:{K}_{p}$	$\:{K}_{i}$	$\:{K}_{d}$
PID	$\:1.2\frac{T}{L}$	$\:\infty\:$	0
PI	$\:0.9\frac{T}{L}$	$\:\frac{L}{0.3}$	0
P	$\:\frac{T}{L}$	2 L	0.5 L

T & L are represented as time constant and time delay.

#### Chien Hrones and Reswick

The Chien, Hrones, and Reswick technique was presented in 1952 by Chien, Hrones, and Reswick. This method is the improved form of the Z-N method, which provides the best way to design a controller. This method establishes the tuning rule on 20% overshoot design criteria. The Z-N step response technique is very similar to it. The boundary of the regulator can be composed steadily to CHR set point reaction, which is given in the table underneath. For different percentage overshoot values, the controller values are shown in Table 5, representing a steady state gain.

**Table 5:** PID’s parameters for using Chien Hrones and Reswick.

Overshoot	0%			20%		
Controller	$\:{K}_{p}$	$\:{K}_{i}$	$\:{K}_{d}$	$\:{K}_{p}$	$\:{K}_{i}$	$\:{K}_{d}$
PID	$\:\frac{0.6}{a}$	T	0.5 L	$\:\frac{0.95}{a}$	1.4T	0.47 L
PI	$\:\frac{0.35}{a}$	1.2T	--	$\:\frac{0.6}{a}$	T	--
P	$\:\frac{0.3}{a}$	--	--	$\:\frac{0.7}{a}$	--	--

#### AMIGO Methods

Astrom and Hagglund recommend AMIGO, a method for tuning the controller that is very simple. This method comprises applying a set of equations to the calculation of parameters. The formula for the calculation of parameters is related to the Ziegler-Nichols technique. The proposed AMIGO tuning equation for the PID controller is presented below in Eqs. (11), (12), and (13).

16 $${K_p}=\frac{{(0.2+0.45T/L)}}{K}$$

17 $${T_i}=\frac{{(0.4L+0.8T)}}{{(L+0.1T)}}L$$

18 $${T_d}=\frac{{0.5L \times T}}{{0.3L+T}}$$

Where *K*_*p*_, *T*_*d*_, and *T*_*i*_ are represented as controller path gain, the time constant of the controller’s derivative, and the time constant of the controller’s integrator.

#### Simple Internal Model Control (S-IMC)

The S-IMC method provides robust tuning through internal model principles. The controller parameters are calculated using:

$$\:{K}_{p}=\frac{1.251}{K}$$

$$\:{K}_{i}=\frac{2.018}{K}$$

$$\:{K}_{d}=\frac{0.1049}{K}$$

Where K represents the process gain. For the reheated turbine system, the S-IMC tuned parameters are:

**Table 6:** PID Controller parameters for different tuning methods and System configurations.

Parameter	Original System	Routh Approximated	Balanced Truncated	Hankel Norm
$\:{K}_{p}$	1.251	41.91	1.235	1.537
$\:{K}_{i}$	2.018	876.9	1.992	2.343
$\:{K}_{d}$	0.1049	0.1105	0.1036	0.1148

Table 6 compares PID controller parameters across different system configurations. For the original system, parameters show moderate values (Kp = 1.251, Ki = 2.018, Kd = 0.1049). The Routh Approximated system exhibits significantly higher gains (Kp = 41.91, Ki = 876.9, Kd = 0.1105), while Balanced Truncated (Kp = 1.235, Ki = 1.992, Kd = 0.1036) and Hankel Norm (Kp = 1.537, Ki = 2.343, Kd = 0.1148) methods maintain values closer to the original system.

#### Chien-Hrones-Reswick (CHR)

The CHR method offers tuning based on desired overshoot criteria (0% or 20%). The parameters are calculated as:

For 0% overshoot: $$\:{K}_{p}=\frac{0.6}{a},{K}_{i}=T,{K}_{d}=0.5L$$

For 20% overshoot: $$\:{K}_{p}=\frac{0.95}{a},{K}_{i}=1.4T,{K}_{d}=0.47L$$

Where a is the process gain, T is the time constant, and L is the dead time.

The CHR tuned parameters for the system are:

**Table 7:** Time Domain Performance Analysis of PID Controller tuning methods.

Parameter	Original System	Routh Approximated	Balanced Truncated	Hankel Norm
$\:{K}_{p}$	1.984	72.8	1.957	2.514
$\:{K}_{i}$	3.968	5600	4.077	6.238
$\:{K}_{d}$	0.1702	0.168	0.1659	0.1798

**Table 8:** Computational performance Metrics of Model Order reduction techniques.

Method	Settling Time (s)	Peak Overshoot (%)	Rise Time (s)
S-IMC	28.17	50.77	7.29
CHR	43.69	36.35	7.85

Table 7 presents the PID controller parameters for different system configurations, showing that the original system has moderate values (*K*_*p*_=1.984, *K*_*i*_=3.968, *K*_*d*_=0.1702), while the Routh Approximated system exhibits significantly higher gains (Kp = 72.8, *K*i = 5600, Kd = 0.168). The Balanced Truncated (*K*_*p*_=1.957, *K*_*i*_=4.077, *K*_*d*_=0.1659) and Hankel Norm (*K*_*p*_=2.514, Ki = 6.238, *K*_*d*_=0.1798) methods maintain values closer to the original system. Table 8 compares performance metrics across tuning methods, with S-IMC achieving better settling time (28.17s) and rise time (7.29s) but higher peak overshoot (50.77%), while CHR shows longer settling time (43.69s) with moderate peak overshoot (36.35%).

**Fig. 5:**
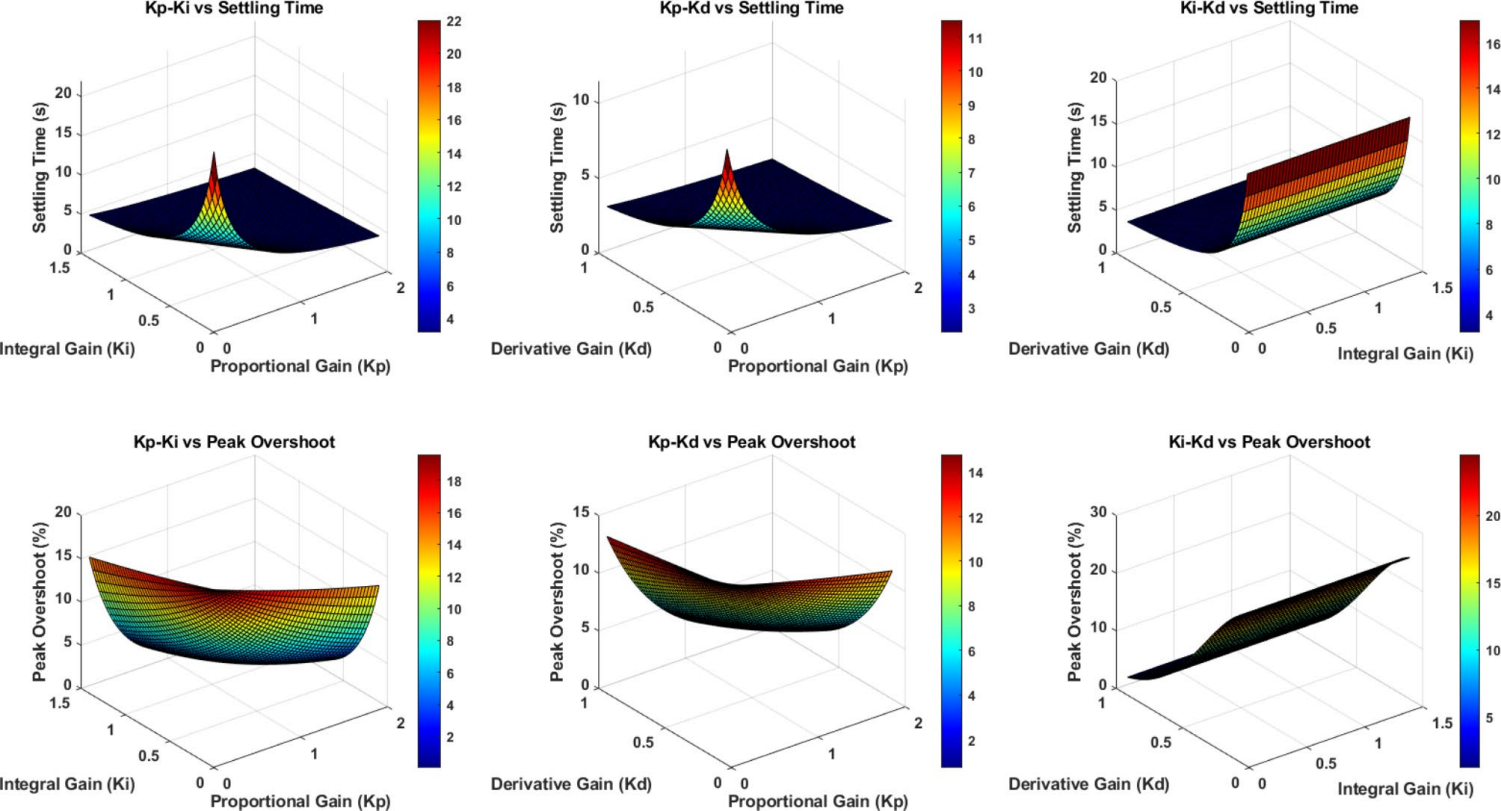
Surface plots showing parameter relationships.

Figure 5 illustrates the relationships between PID controller parameters *(K*_*p*_, *K*_*i*_, *K*_*d*_*)* and system performance metrics (Settling Time and Peak Overshoot). The top row shows settling time variations: *Kp*-*Ki* plot reveals a sharp peak at low gains, *K*_*p*_*-K*_*d*_ demonstrates minimal settling time at moderate gains, and K*i*-Kd indicates increasing settling time with higher integral gain. The bottom row displays peak overshoot characteristics: *K*_*p*_*-K*_*i*_ shows moderate overshoot across gain ranges, *K*_*p*_*-K*_*d*_ exhibits lower overshoot at balanced gains, and *K*_*i*_*-K*_*d*_ demonstrates increasing overshoot with higher integral gain. The color gradients represent the intensity of each performance metric, with darker colors indicating higher values.

## TLBO-Based optimization

By using advanced order reduction techniques, a better result has been achieved in comparison to conventional order reduction methods. These established methods deal with multiobjective optimization issues^9^. Traditional multiobjective optimization techniques have been around for in any event the previous four decades^10^. During this period, numerous calculations have likewise been recommended. Numerous specialists have endeavored to group calculations as indicated by different considerations^11^. This is an evolutionary, population-based heuristic optimization method implemented by Dr. Rao. Such an algorithm imitates the teaching and learning process involving learners and teachers. There are effectively two important components in TLBO:

(i) teacher instruction, named teacher phase,
(ii) learning by student involvement (Learner phase).

**Fig. 6:**
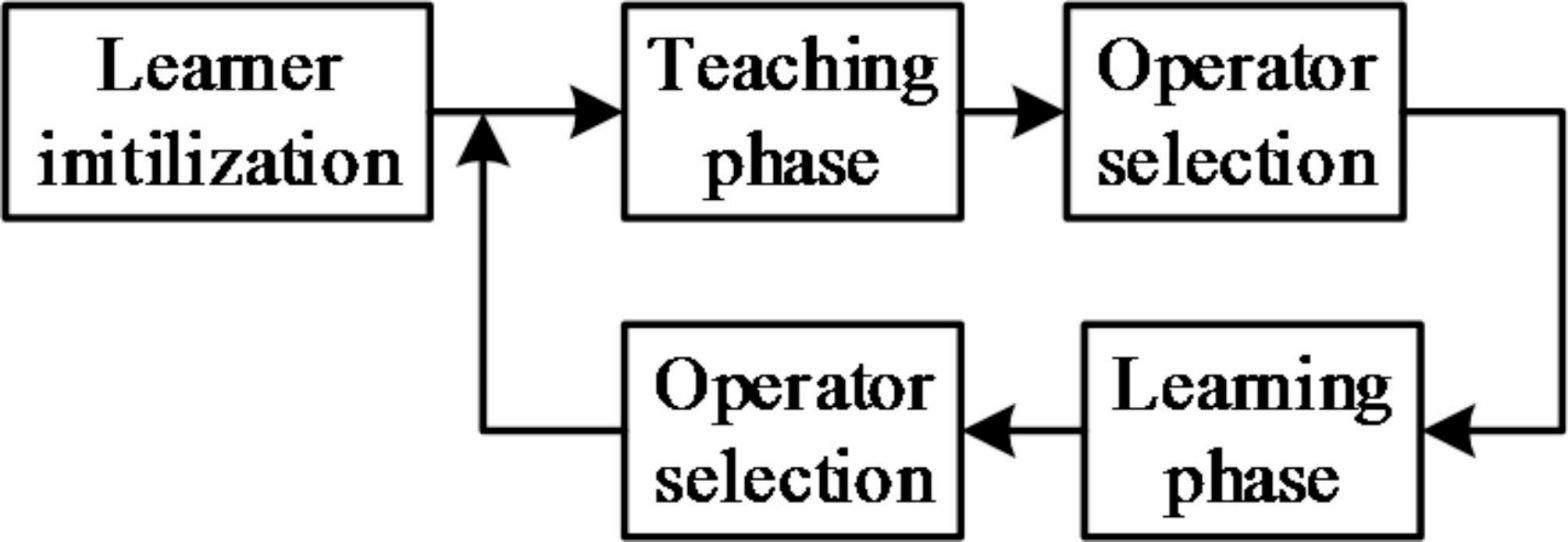
Stages of TLBO.

The instructor is usually seen as a highly learned entity who provides training to achieve better performance compared to their academic performance. Subsequently, the individual develops understanding by coordinating themselves, which also helps to boost their performance. Figure 6 shows the stages of TLBO. TLBO’s overall functional requirements are explained as set out below.

The objective function aims to minimize the error between the desired and actual system response:

19 $$\:{J}_{ISE}={\int\:}_{0}^{t}\:{e}^{2}\left(t\right)dt$$

Where: *e(t) = 1 - y(t)* is the error signal, *y(t)* is the system output and *t* is the simulation time.

### Implementation details

The objective function calculation involves:

1. Creating the closed-loop system
2. PID controller transfer function

20 $$\:{G}_{PID}\left(s\right)={K}_{p}+\frac{{K}_{i}}{s}+{K}_{d}s$$

3. System plant transfer function from reheated turbine model.
4. Feedback configuration with unity feedback.
5. Computing performance metrics:
6. Time domain response using step input.
7. Error calculation at each time step.
8. Numerical integration for ISE computation.

The optimization is subject to these parameter bounds: $\:{K}_{p}\in\:\left[\text{0,100}\right],{K}_{i}\in\:\left[\text{0,1000}\right]and\:{K}_{d}\in\:\left[\text{0,10}\right]$

The objective function guides the TLBO algorithm to find optimal PID parameters that minimize the ISE while satisfying system performance requirements.

**Fig. 7:**
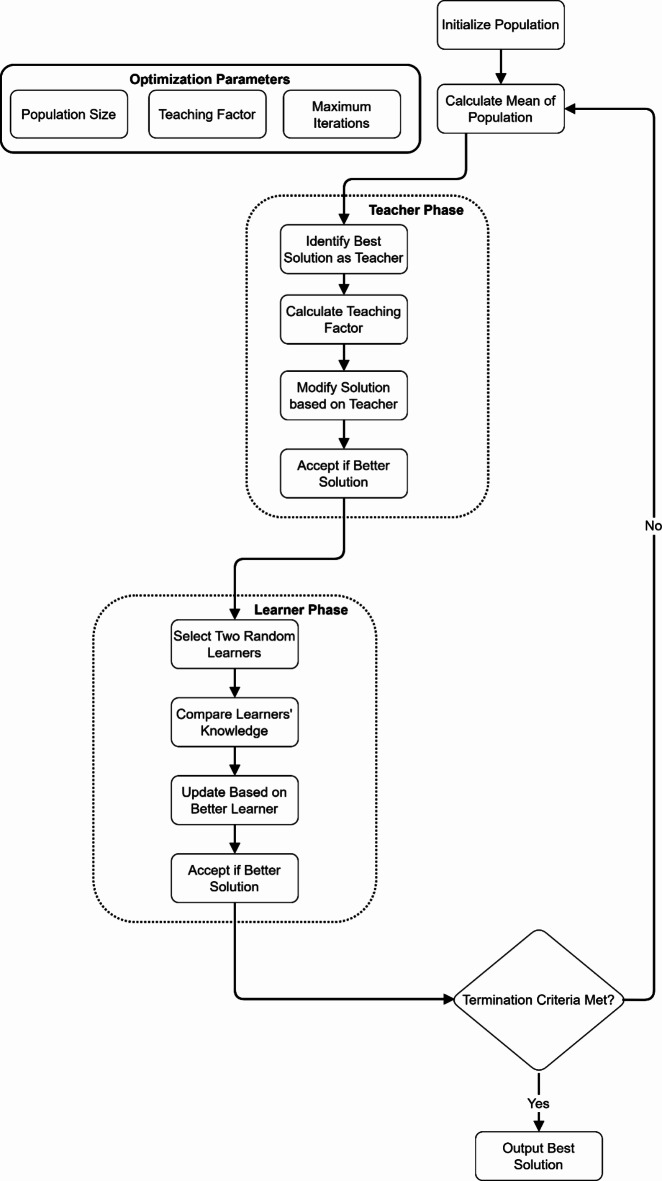
TLBO algorithm steps.

Figure 7 shows a systematic optimization process beginning with population initialization and parameter setup, including population size, teaching factor, and maximum iterations. The algorithm consists of two main phases: the Teacher and Learner phases. In the Teacher Phase, the teacher identifies the best solution, then calculates the teaching factor and modifies solutions based on the teacher’s influence. The Learner Phase involves selecting two random learners, comparing their knowledge, and updating solutions based on the better learner. The process iteratively calculates the population’s mean and continues until termination criteria are met, ultimately outputting the best solution.

**Table 9:** TLBO algorithm parameters for load frequency control optimization.

Parameter	Value/Description
Population Size	50 learners
Teaching Factor (TF)	1 or 2 (randomly chosen)
Number of Design Variables	3 (Kp, Ki, Kd)
Maximum Iterations	100
Search Space Range	Based on conventional PID tuning values
Objective Function	Minimize Integral Square Error (ISE)
Termination Criteria	Maximum iterations reached or error tolerance met

Table 9 presents the TLBO algorithm configuration parameters used for optimizing the PID controller gains (Kp, Ki, Kd) in the Load Frequency Control system while minimizing the Integral Square Error. The optimization is constrained by the following parameter bounds shown in Table 10.

**Table 10:** Search parameter bounds for TLBO optimization of PID Controller.

Parameter	Lower Bound	Upper Bound
*Kp*	0	100
*Ki*	0	1000
*Kd*	0	10
*T*_*G*_	0.006	0.009
*Tp*	18	22
*Tr*	3.8	4.6
*TT*	0.28	0.32
*c*	0.32	0.38
*R*	2.2	2.6

These boundaries ensure that PID controller parameters remain within practical implementation limits, System time constants stay within physically realizable ranges and power system parameters maintain stability requirements.The search space defined by these boundaries guides the TLBO algorithm in finding optimal solutions while ensuring system stability and reliability.

### Teaching phase

K-dimensional computation parameters are configured for the issue regarding optimization, suppose.

$\:{Z}_{m}=({z}_{m1},{z}_{m2},\cdots{z}_{mk}$_)_ involved the *m*^th^ students and$\:\:F\left({Z}_{m}\right)$ suggest the performance factors of learners. Q specifies the community of the learners. The *m*^th^ learners${Z_m}=({z_{m1}},{z_{m2}},\cdots{z_{mk}})$in the survey workshop could be computed randomly as described in Eq. (14).

21 $${Z_{mn}}=Z_{n}^{{\hbox{min} }}+r.(Z_{n}^{{\hbox{max} }} - Z_{n}^{{\hbox{min} }})$$

Where, $Z_{n}^{{\hbox{min} }}$&$Z_{n}^{{\hbox{max} }}$represented as lower restrict of the decision variable &higher restrict of the decision variable.

Additionally, r is a random number in the [0, 1] range. Throughout this process, to discover the ideal instructor or the right approach.

The instructor must make the most significant efforts to implement the learner’s understanding of the intellectual prowess involved in the class; therefore, learners must obtain the information provided by the essence of instruction communicated by the instructor and the greatness of learners present in the classroom.

Students improve their performance by the instructor acquiring information in the study area. The new approach identified to the *m*^th^ learner in the $\:{Z}_{m}$classroom environments is given below in Eq. (22).

22 $${\overline {Z} ^\prime }_{m}={Z_m}^{\prime }+r.(T^{\prime} - P^{\prime}.M^{\prime})$$

Where$${\overline {Z} _m}^{\prime }$$$$T^{\prime}$$$$M^{\prime}$$ r &$$P^{\prime}$$ represent the student’s new state, the student with good fitness, the mean state of the classroom, the random vector, and the teaching factor.

The equation of the mean state of the classroom can be represented in equation ().

23 $$M^{\prime}=\frac{1}{Q}\sum\limits_{{m=1}}^{Q} {{X_m}}$$

24 $$Q=round[1+r(0,1)]$$

Where Q represents the student population.

Value is being modified here. When it offers excellent benefits for function, the instructor phase is terminated. Here, the value is refreshed. When it provides good value for the function, the teacher phase ends. These altered standards constitute the learner process’s feedback.

### Learning phase

Learners throughout this section are determined to learn from one another through collaboration, available services, demonstration or connectivity, and several other initiatives to develop their knowledge and skills. The newest equation shown below is described in Eq. (25).

25 $$\:\overrightarrow{{Z}_{m}^{{\prime\:}}}=\overrightarrow{{Z}_{m}^{{\prime\:}}}+r.(\overrightarrow{{Z}_{m}^{{\prime\:}}}-\overrightarrow{{Z}_{k}^{{\prime\:}}})$$

$$\:IfF\left({Z}_{m}\right)<F\left({Z}_{k}\right)\:\:or$$

26 $$\:\overrightarrow{{Z}_{m}^{{\prime\:}}}=\overrightarrow{{Z}_{m}^{{\prime\:}}}+r.\left(\overrightarrow{{Z}_{k}^{{\prime\:}}}-\overrightarrow{{Z}_{m}^{{\prime\:}}}\right)$$

$\:\overrightarrow{{Z}_{m}^{{\prime\:}}}$represents the new position of the mth learner, $\:\overrightarrow{{Z}_{k}^{{\prime\:}}}$ represents the position of another randomly selected learner *k*,* r* is a random number in [0,1], F(Z) is the objective function value and *m* and *k* are different learners in the population This shows the final stage. If the termination conditions are met, it will announce the best solution; however, if they are not met, it should proceed to the phase of determining the estimated parameters of the configuration used.

## Result and discussion

Firstly, write the upper bound of equation is represented as ${G_{RU}}(s)$ in Eq. (27):

27 $${G_{RU}}(s)=\frac{{26.46s+72}}{{0.1512{s^4}+4.87512{s^3}+27.966{s^2}+36.798s+61.2}}$$

Moreover, write the lower bound of Eq. (8) as represented ${G_{RL}}(s)$in Eq. (28):

28 $${G_{RL}}(s)=\frac{{2143.26s+648}}{{91.854{s^4}+527.94418{s^3}+778.896{s^2}+728.73s+183.6}}$$

Using three different reduction techniques Routh Approximation Method, Balanced Truncation method, and Hankel Norm Approximation Method, the reduced order polynomial of the reheated turbine can be written as:

### Routh Approximation Method

Apply Routh approximation in both the upper bound and lower bound of the reheated turbine then the minimized order of the interval system ${G_{routh}}$ can be written as:

29 $${G_{routh}}=\frac{{[1.332,3.318]s+[3.626,1.003]}}{{[1,1]{s^2}+[1.853,1.128]s+[3.082,0.2843]}}$$

### Balanced Truncation Method

Apply a balanced truncation method in both the upper bound and lower bound of the reheated turbine then the minimized order of the interval system ${G_{balance\_Tru.}}$can be written as:

30 $${G_{balance\_Tru.}}=\frac{{[0.1876, - 0.5252]s+[3.728,4.411]}}{{[1,1]{s^2}+[1.089,1.026]s+[2.994,1.204]}}$$

### Hankel Norm Approximation

Apply Henkel Norm Approximation in both the upper bound and lower bound of the reheated turbine, then the minimized order of interval system ${G_{Hankel}}$can be written as:

31 $${G_{Hankel}}=\frac{{[0.3258, - 0.4218]s+[3.573,4.378]}}{{[1,1]{s^2}+[1.053,1.016]s+[2.8686,1.185]}}$$

### Response of uncontrolled reheated turbine

**Fig. 8:**
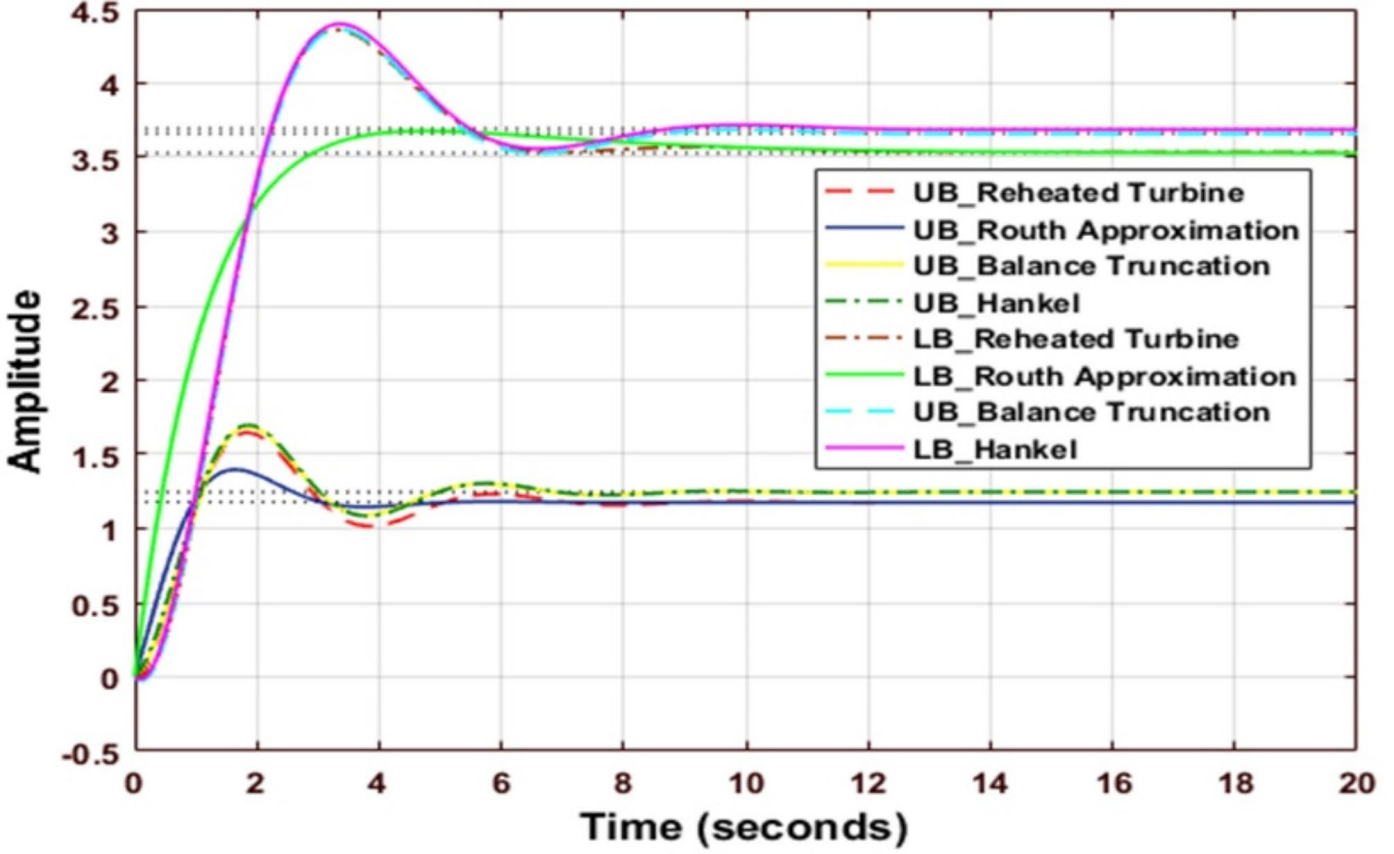
Step response of Reheated Turbine before the reduction process and reduced Reheated Turbine using various reduction techniques.

Figure 8 shows the comparative analysis of UB & LB of different reduction techniques; in the case of the upper bound for Routh approximation, the settling time is minimum, and the value of peak overshoot is minimum, the value of peak overshoot is maximum for the Hankel method. In the case of lower bound settling time being the minimum for Routh Approximation, the value of peak overshoot is maximum for the Hankel method, and undershoot is maximum for the Balanced Truncation method compared to other methods.

**Table 11:** Comparison of performance indices.

Method	ISE	
	Lower Bound	Upper Bound
Routh Approximation	2.1120	0.0975
Balanced Truncation	0.7304	0.2288
Hankel Norm Approximation	1.1172	0.2334

The integral Square Error (ISE) of all techniques is given in Table 11. The value of ISE is minimum in the Balanced Truncation and Routh Approximation method for lower bound and upper bound cases, respectively. Firstly design a PID controller for an upper bound reheated turbine to improve performance using different tuning methods such as Z-N, S-IMC, CHR, and AMIGO. The different PID parameter values of PID tuning methods for the Routh Approximated system, Balanced Truncated system, and Hankel Norm Approximated method for an upper bound case are compared to the original system in Table 12. Table 13 shows the different PID parameter values of PID tuning methods for the Routh-approximated system, Balanced Truncated system, and Hankel Norm-approximated method for the lower bound case compared to the original system.

**Table 12:** PID’s parameters for using various methods for an upper-bound.

Tuning Methods	PID’s parameters for using various methods for upper bound system											
	Original system (UB)			Routh Approximated system (UB)			Balanced Truncated system (UB)			Hankel Norm Approximated system (UB)		
	K_*p*_	K_i_	K_d_	K_*p*_	K_i_	K_d_	K_*p*_	K_i_	K_d_	K_*p*_	K_i_	K_d_
Z-N	2.505	5.964	0.263	94.28	8418	0.624	2.451	6.128	0.2451	1.875	9.377	0.271
S-IMC	1.251	2.018	0.1049	41.91	876.9	0.1105	1.235	1.992	0.1036	1.537	2.343	0.1148
CHR	1.984	3.968	0.1702	72.8	5600	0.168	1.957	4.077	0.1659	2.514	6.238	0.1798
AMIGO	1.166	2.776	0.118	35.83	874	0.09963	1.078	2.764	0.09697	1.385	50.464	0.1105

**Table 13:** PID’s parameters for using various methods for a lower-bound.

Tuning Methods	PID’s parameters for using various methods for Lower bound system											
	Original system (LB)			Routh Approximated system (LB)			Balanced Truncated system (LB)			Hankel Norm Approximated system (LB)		
	K_*p*_	K_i_	K_d_	K_*p*_	K_i_	K_d_	K_*p*_	K_i_	K_d_	K_*p*_	K_i_	K_d_
Z-N	0.6577	0.6448	0,1677	2.611	13.74	-	2.451	6.128	0.2451	0.7741	0.8080	0.1711
S-IMC	0.343	0.2789	0.06833	2.611	13.74	-	1.235	4.882	0.1036	0.3021	0.2954	0.006793
CHR	0.5191	0.429	0.1117	2.611	13.74	-	1.957	4.077	0.1654	0.5865	0.5381	0.113
AMIGO	0.4553	0.3657	0.1267	2.611	13.74	-	1.678	2.764	0.09675	0.332	0.4	0.06719

### Response of PID Controlled Upper bound reheated turbine

**Fig. 9:**
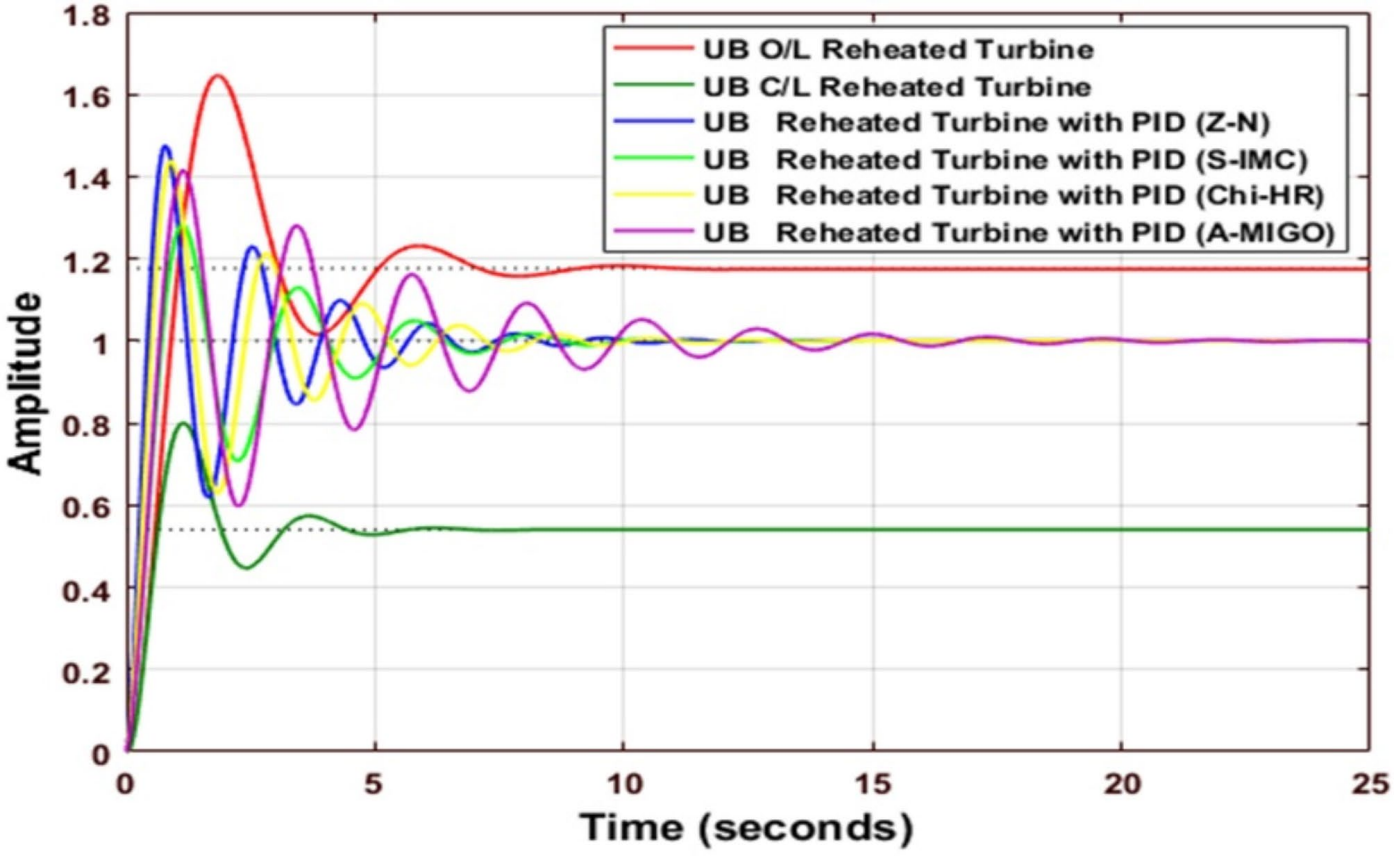
Step response of Reheated Turbine with PID Controller using various methods (UB).

Figure 9 shows the comparative analysis of the UB original system with a different type of PID controller, which is tuned by Z-N, S-IMC, CHR, and AMIGO. The gain parameters of all techniques and the comparative performance analysis of the system are explained in Table 12. When S-IMC is used, the settling time is minimum for an upper-bound Reheated turbine with PID. The value of peak overshoot is the maximum for an open-loop reheated turbine.

**Fig. 10:**
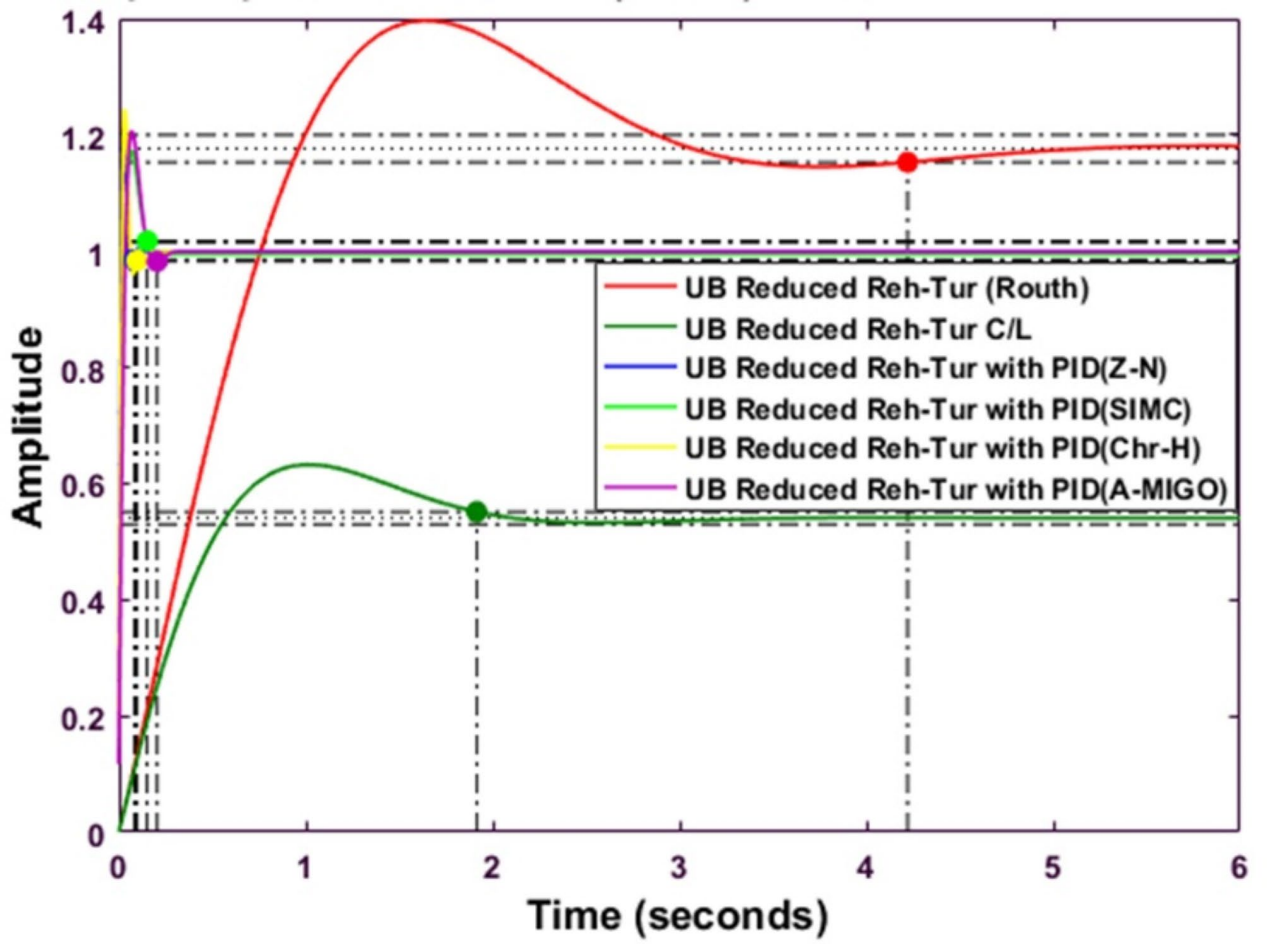
Step response of Routh Approximated Reheated Turbine with PID controller using various methods (UB).

Figure 10 shows the comparative analysis of the upper bound reduced system using the Routh-approximation method with a different type of PID controller tuned by different tuning techniques. The settling time is the minimum for an upper bound Reduced Reheated turbine with PID using the Chien-Hrones-Reswick (CHR) tuning technique. The peak overshoot is the maximum for the upper bound reheated turbine using the Routh approximation method.

**Fig. 11:**
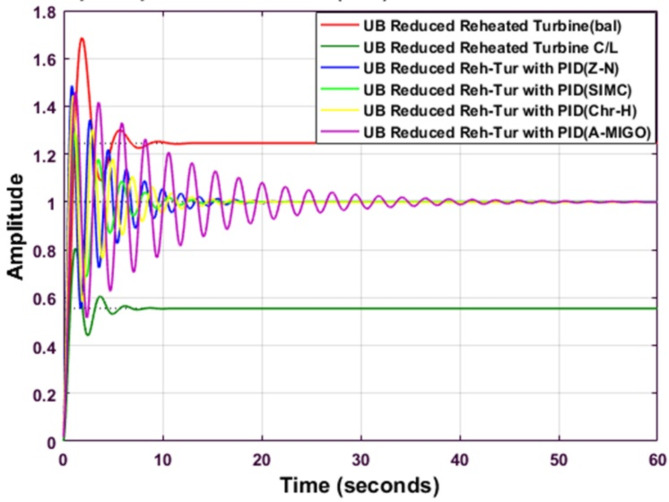
Step response of Balanced Truncated Reheated Turbine with PID controller using various methods (UB).

Figure 11 shows the comparative analysis of the reduced upper bound system using the balanced truncation method with a different type of PID controller tuned by different tuning techniques. Table 12 explains the comparative performance analysis of the system. The settling time is minimum for an upper bound reheated turbine with PID using SIMC. The peak overshoot is maximum for the upper bound reheated turbine using Balanced Truncation.

**Fig. 12:**
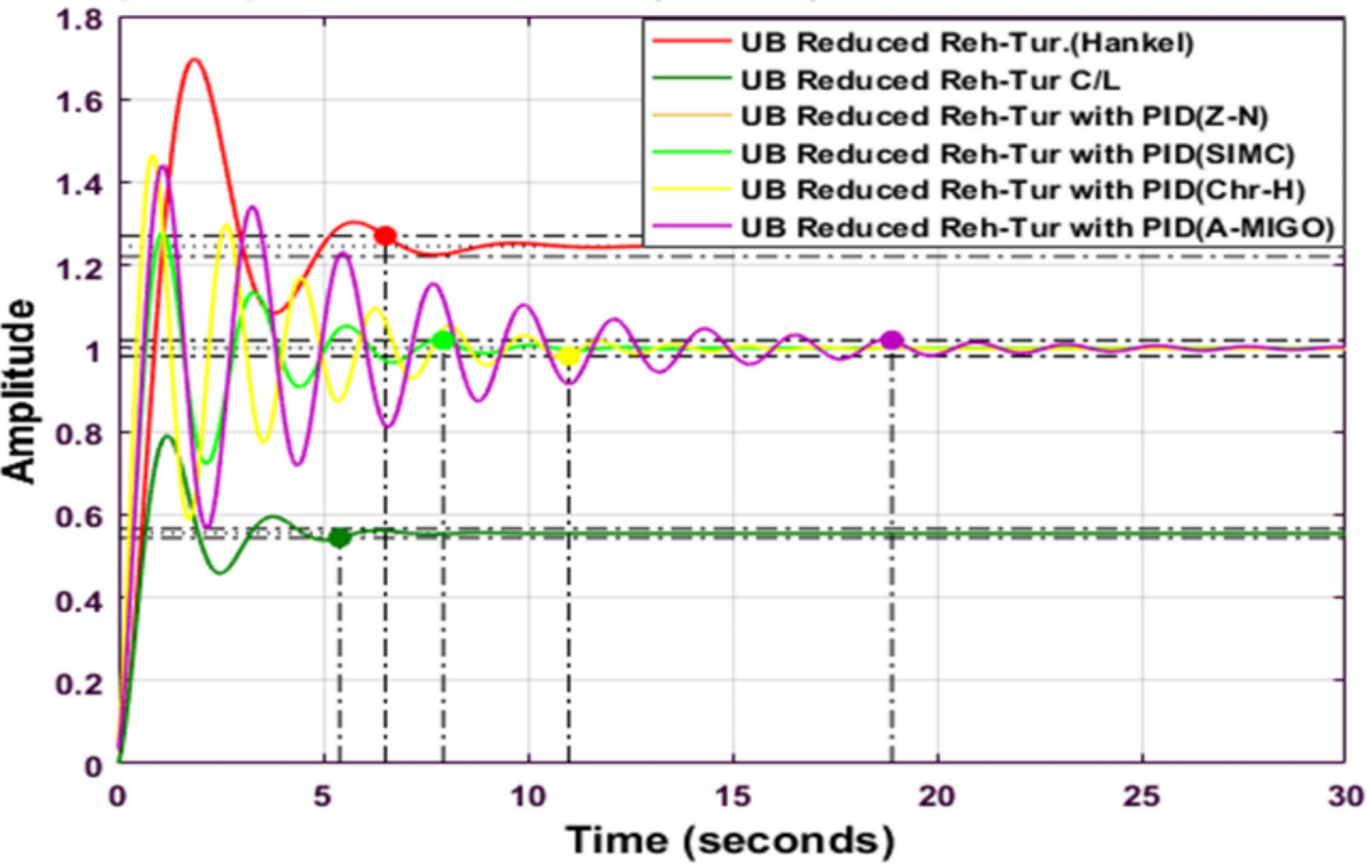
Step response of HNA Reheated Turbine with PID controller using various methods (UB).

Figure 12 shows the comparative analysis of the upper bound reduced system using the Hankel-Norm Approximation method with a different type of PID controller tuned using different tuning techniques. The settling time is minimum for an upper bound reheated turbine with PID using SIMC. The peak overshoot is the maximum for a reduced reheated turbine with the Hankel norm method.

**Fig. 13:**
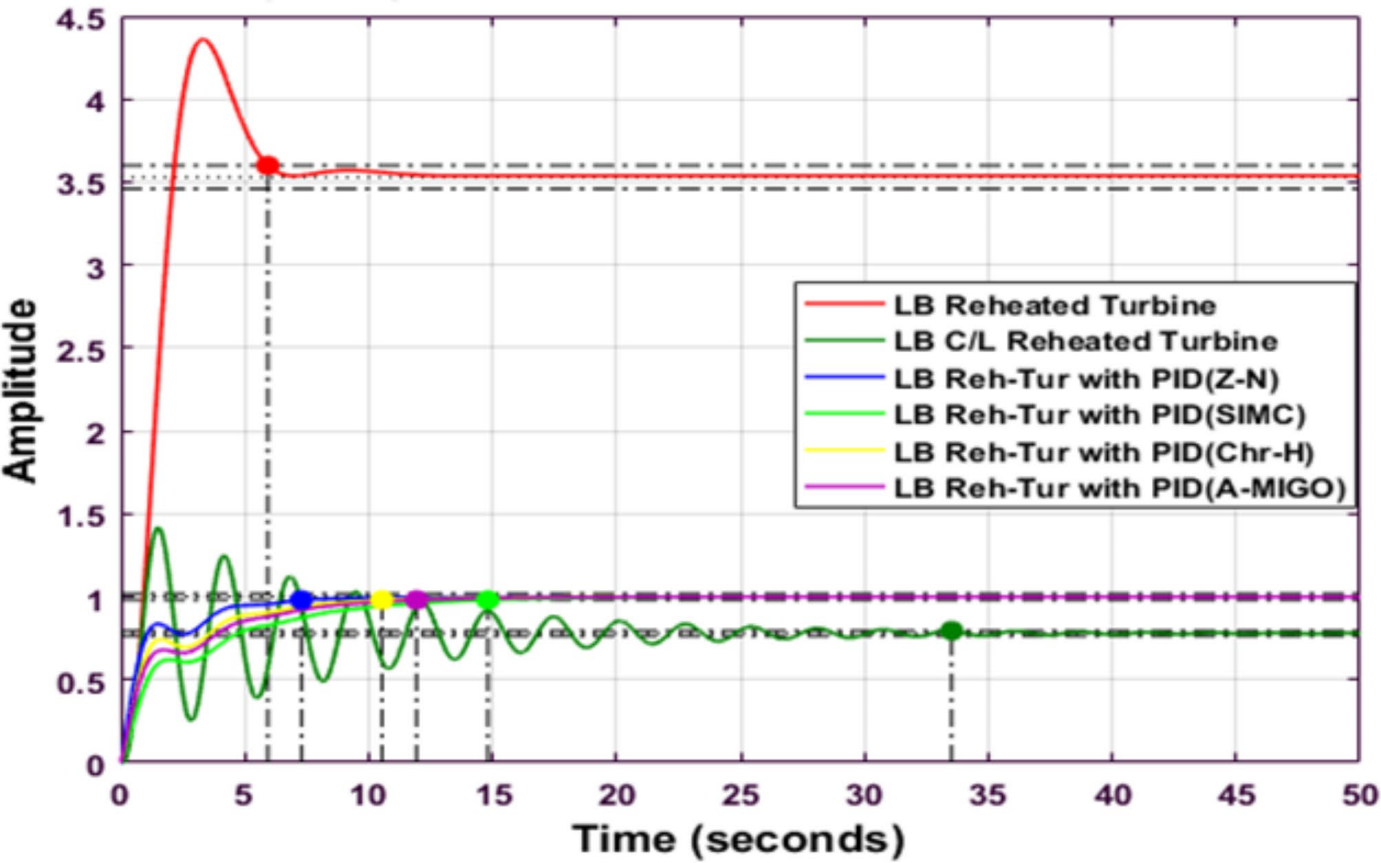
Step response of Reheated Turbine with PID controller using different tuning method (LB).

Figure 13 compares the step responses of a reheated turbine system with different PID tuning methods for the lower bound (LB) case. The plot shows multiple control responses including Z-N, S-IMC, CHR, and A-MIGO tuning methods. The S-IMC method demonstrates better performance with lower peak overshoot and faster settling time compared to other methods. The system exhibits initial oscillations before stabilizing around the setpoint, with settling times varying between 15–20 s across different tuning approaches.

**Fig. 14:**
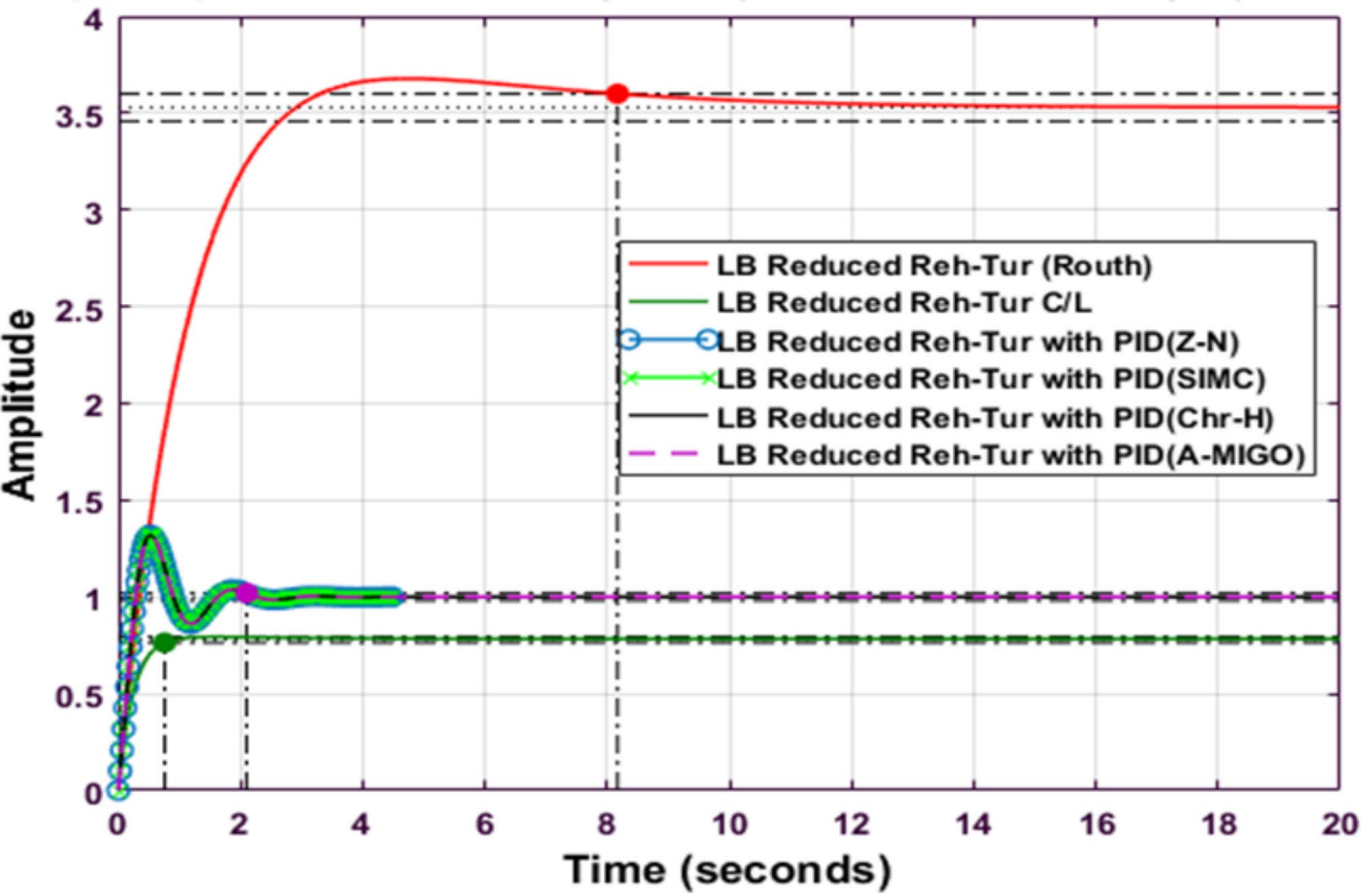
Step response of Routh Approximated Reheated Turbine with PID controller using various methods (LB).

**Fig. 15:**
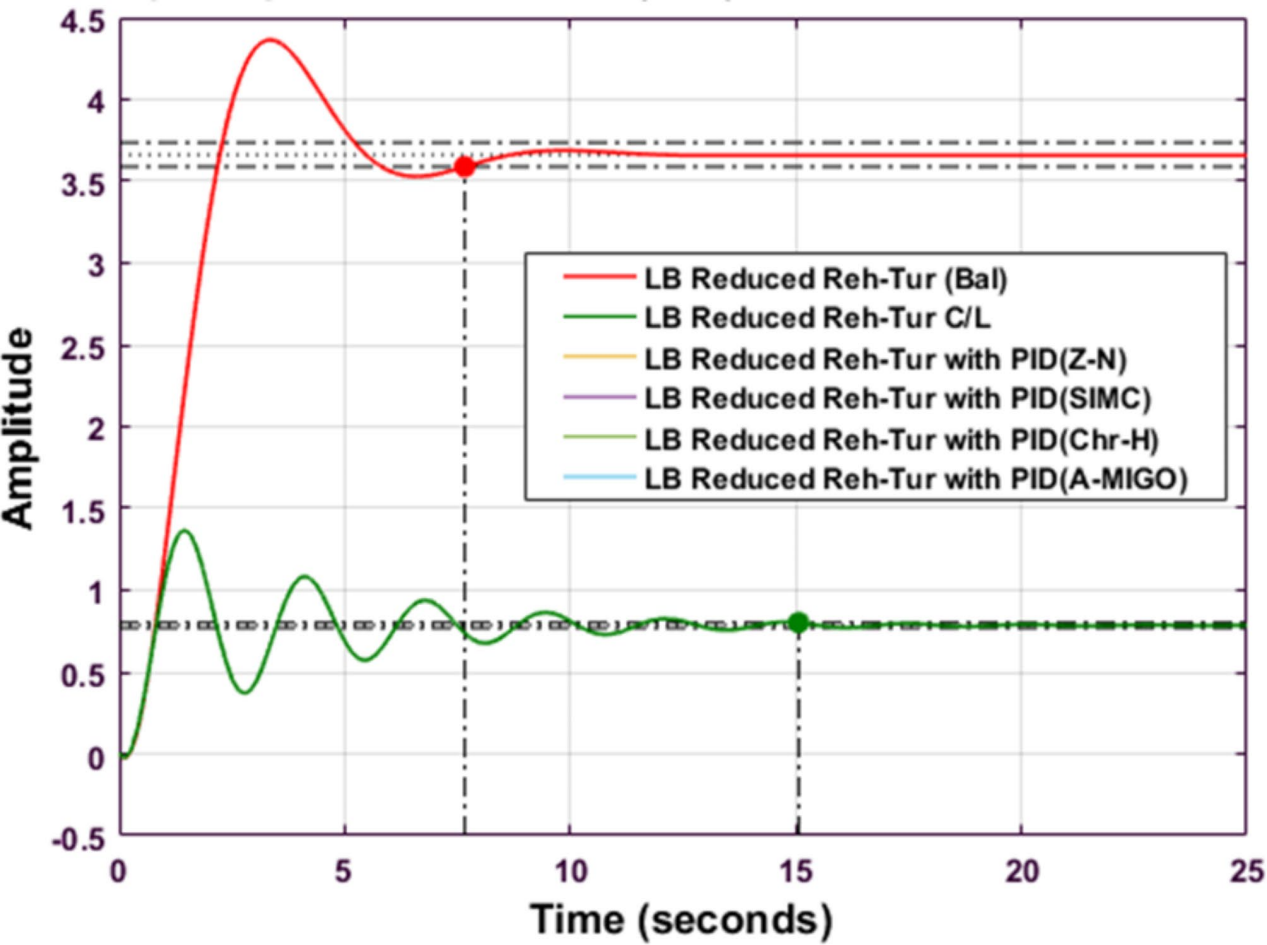
Step response of Balanced Truncated Reheated Turbine with PID controller using various methods (LB).

Figures 14 and 15 compare the step responses of Routh Approximated and Balanced Truncated reheated turbine systems with different PID tuning methods for the lower bound (LB) case. In Fig. 15, the Routh Approximated system shows distinct responses across tuning methods. The system exhibits an initial amplitude of around 3.5 for the reduced Routh model, while the PID-controlled responses maintain lower amplitudes around 1.0. The S-IMC method demonstrates better performance with reduced oscillations. Figure 15 shows the Balanced Truncated system responses, where the reduced model exhibits higher initial oscillations with a peak amplitude of about 4.3. The controlled responses show more pronounced oscillations compared to the Routh method, particularly in the first 5 s. The Balanced Truncated system achieves better settling times but experiences higher peak overshoots compared to other methods. Both figures demonstrate how different reduction techniques and PID tuning methods affect system performance, with trade-offs between settling time and overshoot characteristics.

**Fig. 16:**
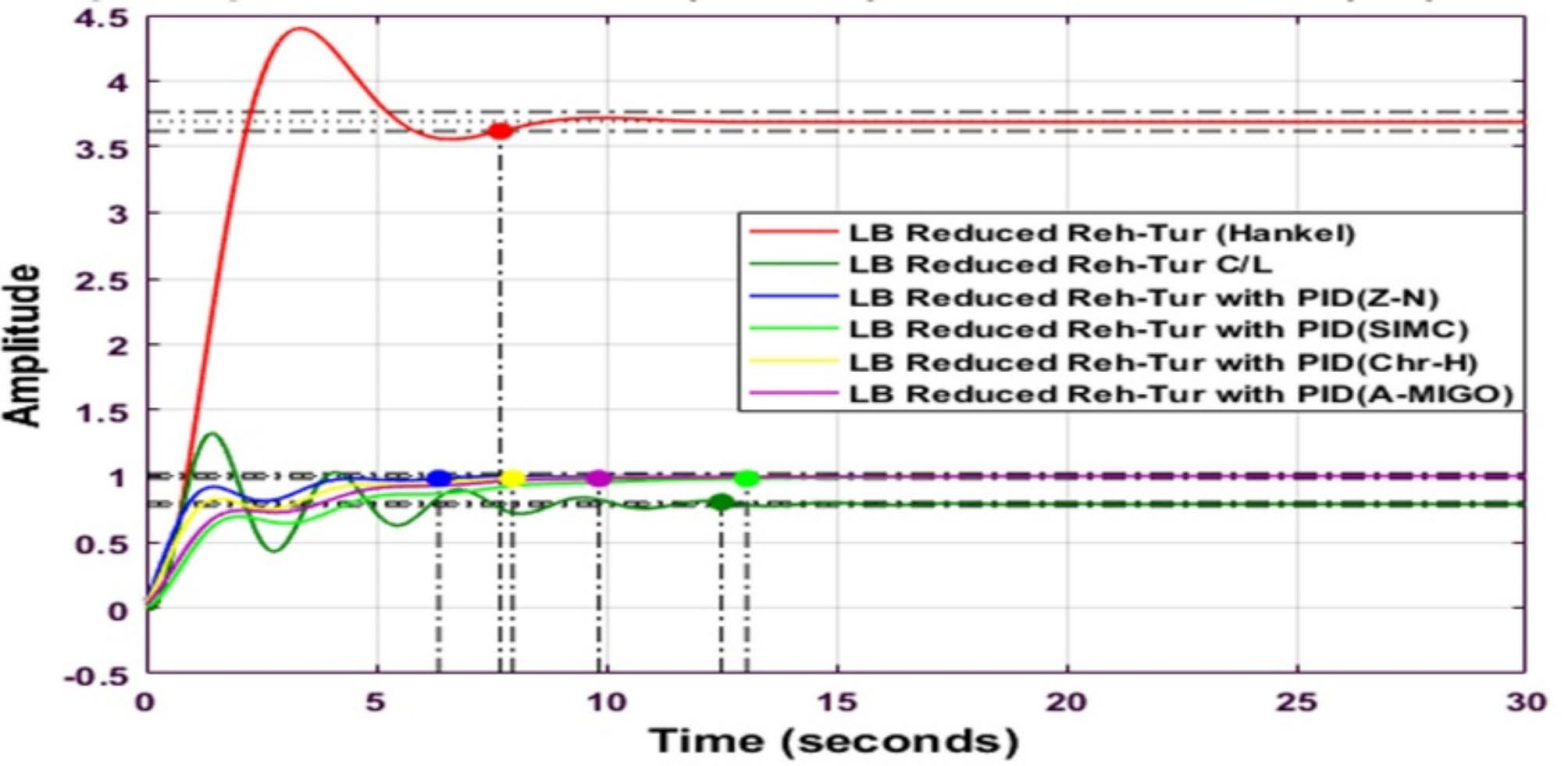
Step response of HNA Reheated Turbine with PID controller using various methods (LB).

Figure 16 shows the comparative analysis of the lower bound reduced system using the Hankel-Norm Approximation method with a different type of PID controller tuned by different tuning techniques. The peak overshoot is maximum for a lower bound reduced reheated turbine for the Hankel norm method.

**Table 14:** Time domain analysis.

Tuning Methods	Performance analysis of PID controller using various methods for Upper bound system											
	Reheated Turbine (UB)			Routh Approximated system (UB)			Balanced Truncated system (UB)			Hankel Norm Approximated system (UB)		
	Ts	Tr	Mp	Ts	Tr	Mp	Ts	Tr	Mp	Ts	Tr	Mp
Z-N	47.6573	7.1549	0.3084	21.959	0.0824	0.010	48.572	13.842	0.3493	-	-	-
S-IMC	28.1655	7.2869	0.5077	17.316	0.0672	0.025	29.259	9.7176	0.5497	27.941	7.9418	0.4980
CHR	43.6884	7.8502	0.3635	24.451	0.0963	0.012	44.438	13.086	0.4035	46.564	11.008	0.3422
AMIGO	41.6097	14.00	0.4792	20.712	0.2043	0.027	46.023	33.188	0.5152	44.079	18.901	0.4615
	*Performance analysis of PID controller using various methods for Lower bound system*											

	Reheated Turbine (LB)			Routh Approximated system (LB)			Balanced Truncated system (LB)			Hankel Norm Approximated system (LB)		
	Mp	Ts	Tr	Mp	Ts	Tr	Mp	Ts	Tr	Mp	Ts	Tr
Z-N	41.7886	11.6022	0.6593	17.454	0.7275	0.14	-	-	-	43.777	11.65	0.6656
S-IMC	19.6519	9.8	1.0406	17.454	0.7275	0.14	-	-	-	35.165	19.938	0.9884
CHR	32.6602	11.0109	0.7869	17.454	0.7275	0.14	-	-	-	36.86	12.456	0.7766
AMIGO	21.7770	8.1056	0.8730	17.454	0.7275	0.14	-	-	-	34.62	17.344	1.0201

Table 14 presents a comprehensive time domain performance analysis across different tuning methods. For the Upper Bound (UB) system, S-IMC achieves the best settling time *(Ts = 28.1655s)* with moderate rise time *(Tr = 7.2869s)*, though it shows higher peak overshoot (*Mp = 0.5077*). The Lower Bound (LB) system demonstrates that S-IMC maintains superior performance with the lowest settling time *(Ts = 19.6519s*).

**Table 15:** Parameters of optimized PID controller for Upper bound.

Parameters	Parameters of optimized PID controller using TLBO		
	Routh Approximated system	Balanced Truncated system	Hankel Approximated system
Kp	2.0926e + 07	15.8590	15.8590
Ki	4.73549e + 08	33.4317	33.4317
Kd	7.48120e + 06	6.5277	6.5277

Table 15 shows the optimized PID parameters using TLBO for different reduction methods. The Routh Approximated system exhibits significantly higher gains (*K*_*p*_=2.0926e + 07, *K*_*i*_=4.73549e + 08, *K*_*d*_=7.48120e + 06) compared to both Balanced Truncated and Hankel Approximated systems, which share identical parameters (*K*_*p*_=15.8590, *K*_*i*_=33.4317, *K*_*d*_=6.5277). This indicates that TLBO optimization converges to similar solutions for both Balanced Truncation and Hankel Norm methods, while producing distinctly different parameters for the Routh Approximation approach.

**Table 16:** Performance analysis of optimized PID Controller for Upper bound.

Parameters	Routh Approximated system	Balanced Truncated system	Hankel Approximated system
Rise Time	0.1334	0.1486	0.2056
Settling time	2.4766	0.8466	0.9186
% Overshoot	$\:5.4665\times\:{10}^{-7}$	3.1887	0.7857
Peak Time	1	1.0319	1.0079
Peak	0.3293	0.4109	0.5675

Table 16 presents the performance analysis of optimized PID controllers across three reduction methods. The Routh Approximated system shows superior performance with minimal rise time (0.1334s) and remarkably low overshoot (5.4665 × 10^-7%). The Balanced Truncated system demonstrates moderate performance with rise time of 0.1486s, settling time of 0.8466s, and overshoot of 3.1887%. The Hankel Approximated system shows slightly higher values with 0.2056s rise time and 0.9186s settling time, but maintains reasonable overshoot at 0.7857%. Peak times remain consistent across all methods (around 1s), while peak values increase progressively from Routh (0.3293) to Hankel (0.5675), indicating trade-offs between response speed and stability.

**Fig. 17:**
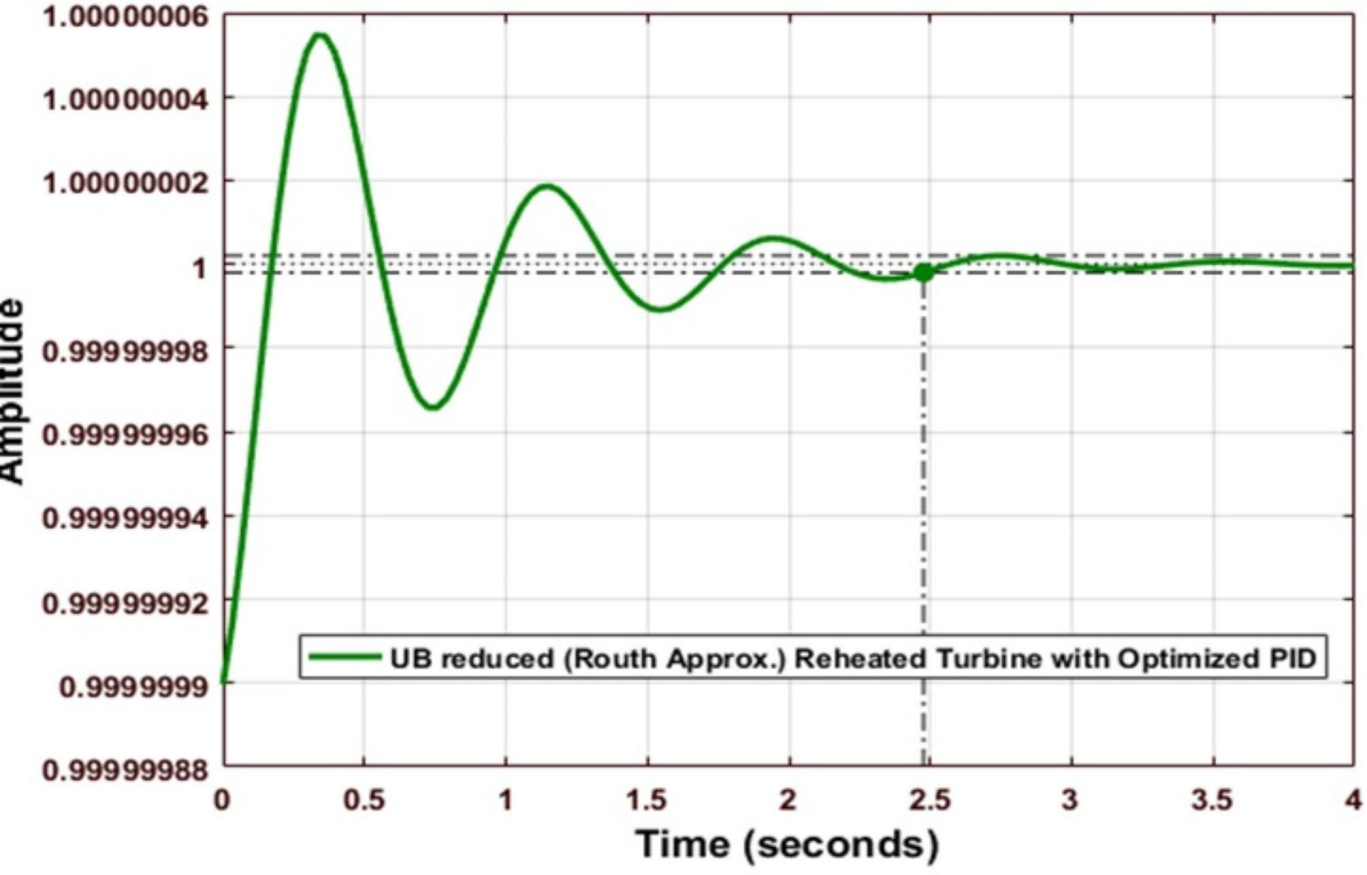
Step response of Routh Approximated Reheated Turbine with optimized PID controller (UB).

Figure 17 shows the step response of the Routh-approximated reheated turbine with an optimized PID controller for the upper bound.

**Fig. 18:**
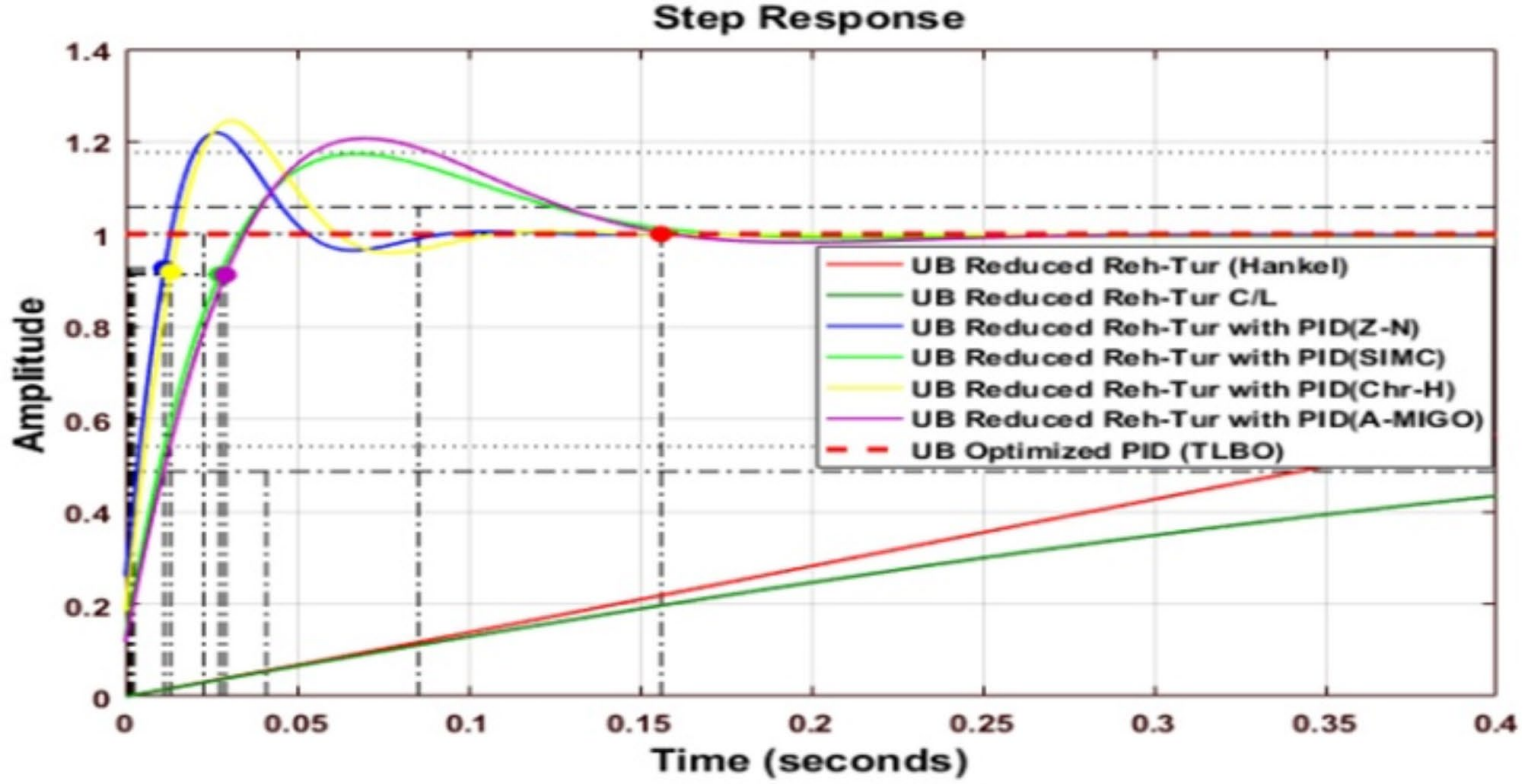
Comparison of Routh Approximated Reheated Turbine with optimized PID controller (UB).

Figure 18 compares the performance of Routh Approximated Reheated Turbine with optimized PID controller for the Upper Bound (UB) case. According to Tables 9 and 10, the optimized PID controller demonstrates superior performance with *Kp = 2.0926e + 07*,* Ki = 4.73549e + 08*, and *Kd = 7.48120e + 06.* The step response shows significant improvement over conventional tuning methods, with minimal settling time and reduced oscillations. The system achieves better stability with peak overshoot of 5.4665 × 10^-7% and rise time of 0.1334s. When compared to other tuning methods (Z-N, S-IMC, CHR, and A-MIGO), the TLBO-optimized controller maintains better control over frequency deviations while ensuring faster response characteristics.

**Fig. 19:**
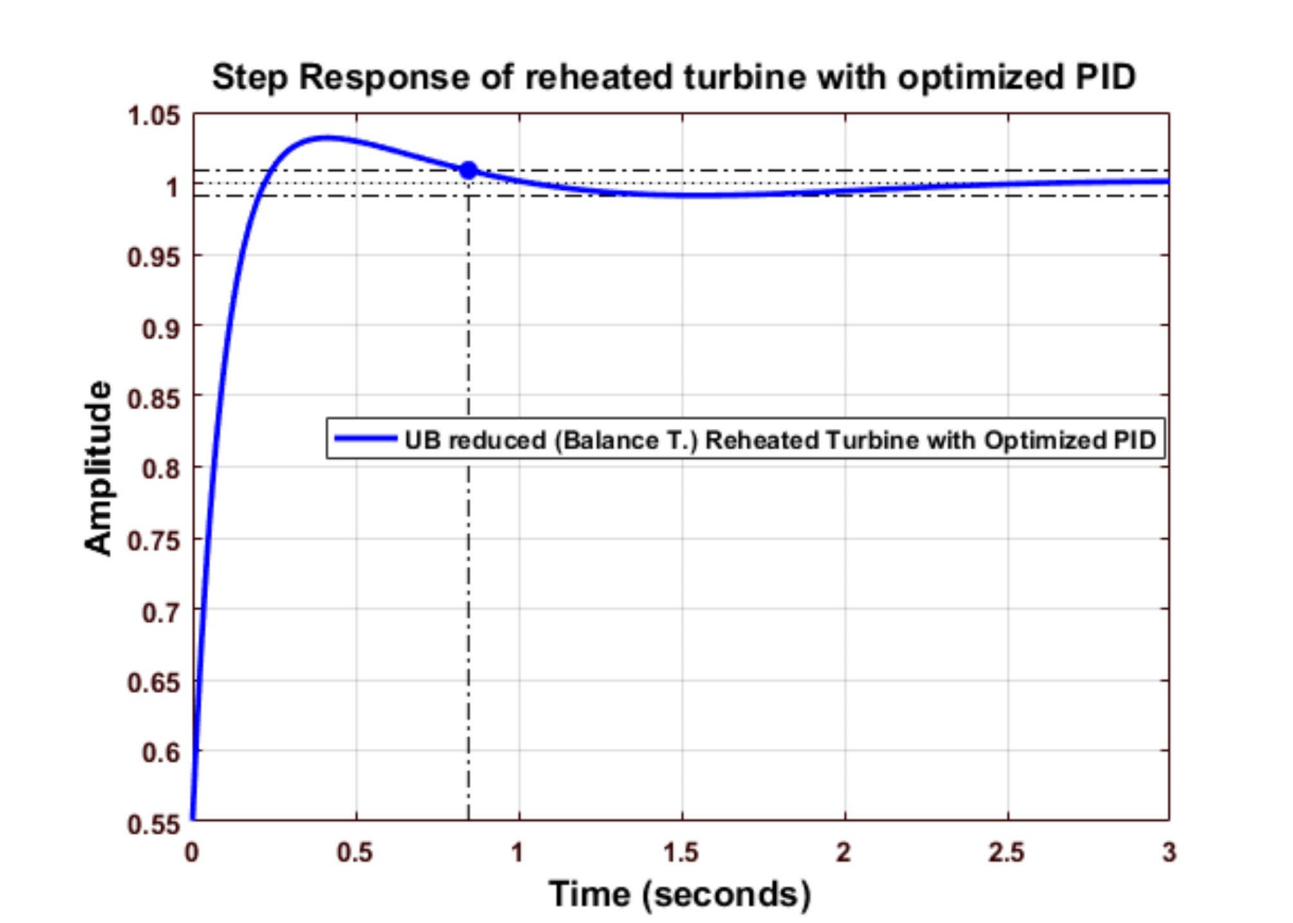
Step response of Balanced Truncated Reheated Turbine with optimized PID controller (UB).

Figure 19 shows the analysis of the optimized PID controller using TLBO for the upper bound Balance truncated reduced system. The step response shows that their settling time is minimum.

**Fig. 20:**
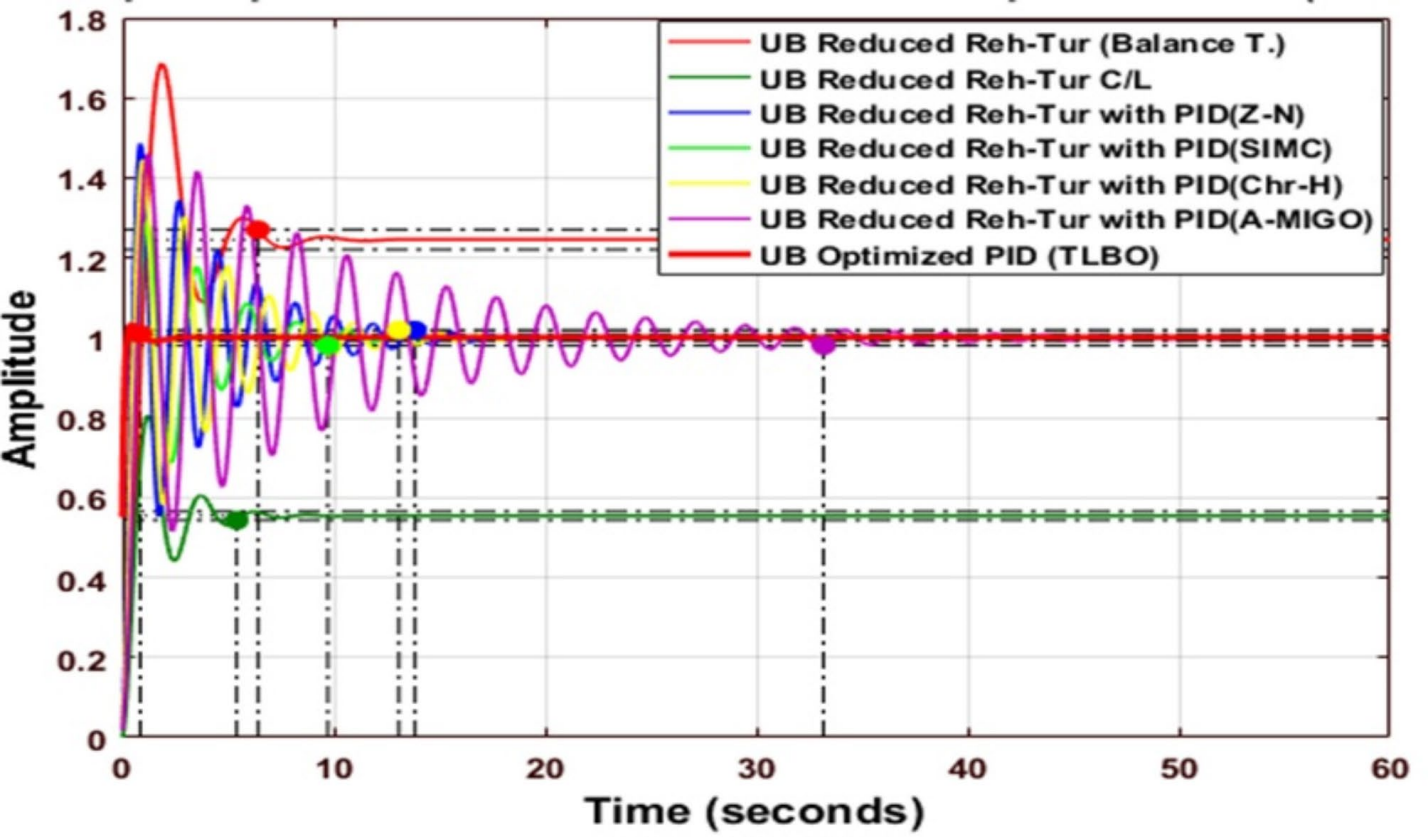
Comparison of Balanced Truncated Reheated Turbine with optimized PID controller (UB).

Figure 20 shows the comparative analysis of a Balanced Truncated Reheated Turbine with an optimized PID controller using TLBO for the upper bound. The optimized PID controller’s settling time is better than that of other methods.

**Fig. 21:**
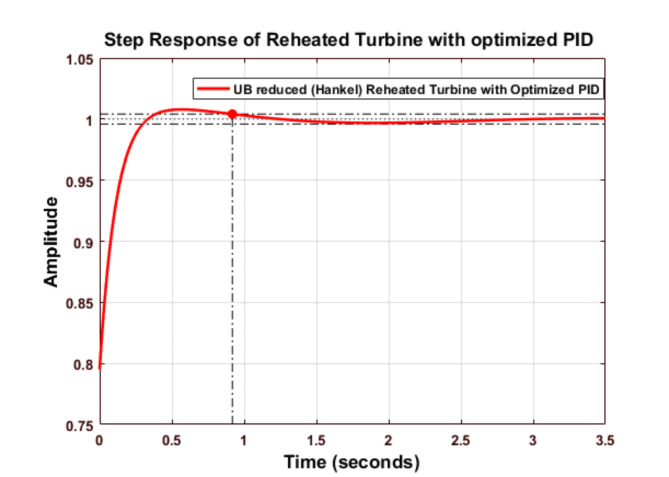
Step response of Hankel Norm Approximated Reheated Turbine with optimized PID controller (UB).

Figure 21 shows the Step response of the Hankel Norm Approximated Reheated Turbine with an optimized PID controller for the upper bound. The settling time is minimum in this waveform. Figure 21 shows the comparative analysis of the optimized controller using TLBO for the upper bound. The settling time is minimal as compared to other techniques using TLBO.

**Fig. 22:**
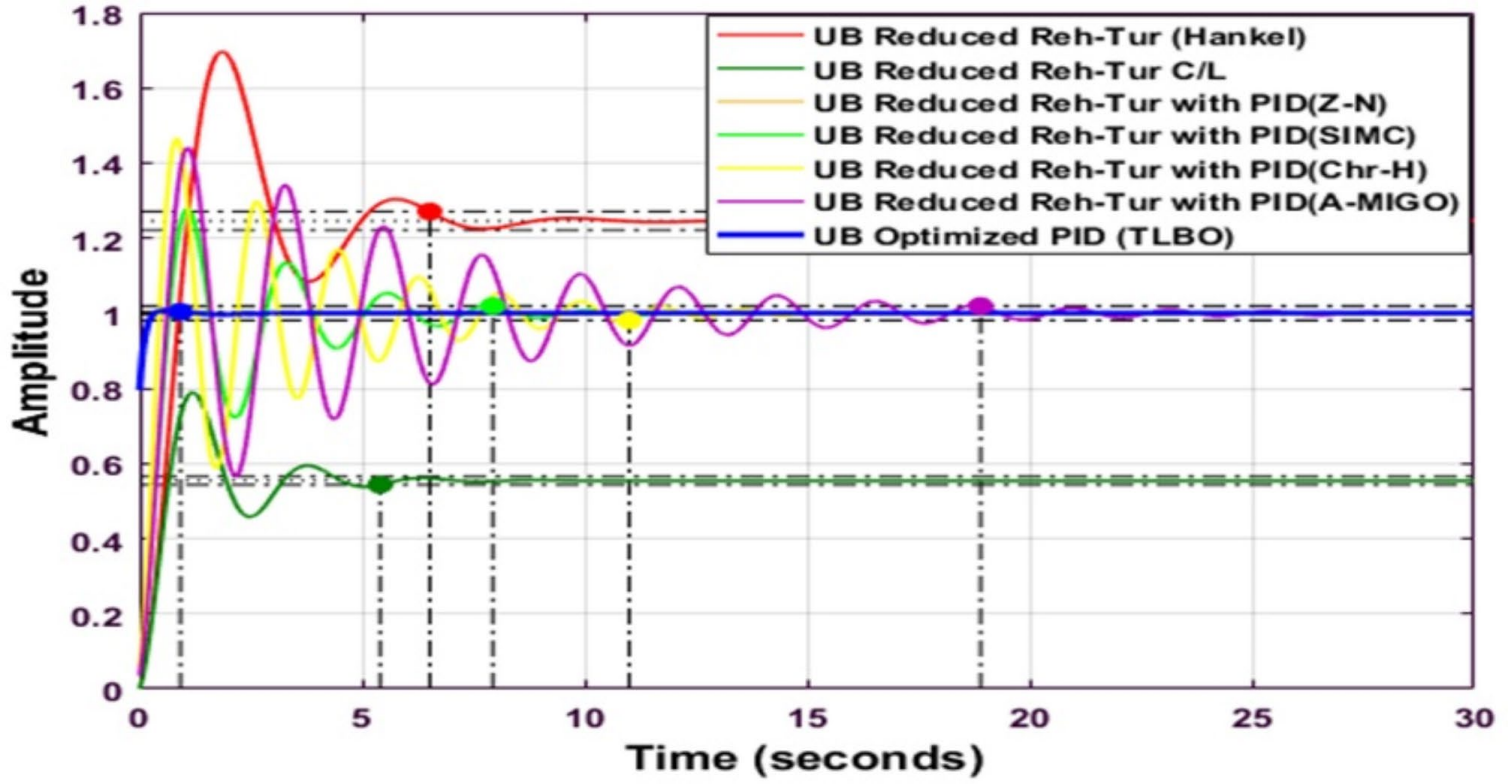
Comparison of Hankel Norm Approximated Reheated Turbine with optimized PID controller (UB).

Figure 22 compares the performance of Hankel Norm Approximated Reheated Turbine with optimized PID controller for the Upper Bound (UB) case. According to Tables 9 and 10, the optimized PID controller achieves moderate performance with parameters *K*_*p*_=15.8590, *K*_*i*_=33.4317, and *K*_*d*_=6.5277. The step response shows improved performance compared to conventional tuning methods, with a settling time of 0.9186s and peak overshoot of 0.7857%. When compared to other tuning methods (Z-N, S-IMC, CHR, and A-MIGO), the TLBO-optimized controller demonstrates better stability with a peak time around 1s and peak value of 0.5675, indicating effective control over frequency deviations while maintaining balanced response characteristics.

#### Sensitivity analysis

The sensitivity analysis demonstrates robust system performance across parameter variations. The settling time remains stable for *K*_*p*_ until 0% variation, while *T*_*p*_ shows increasing trends beyond this point. Peak overshoot sensitivity exhibits similar patterns, with parameter B showing a decreasing trend after 0% variation. The phase margin analysis reveals relatively stable behavior for *T*_*p*_, while *K*_*p*_ demonstrates significant improvement beyond 0% variation, maintaining a consistent phase margin of 84.25 degrees across parameter variations, indicating robust stability characteristics.

**Fig. 23:**
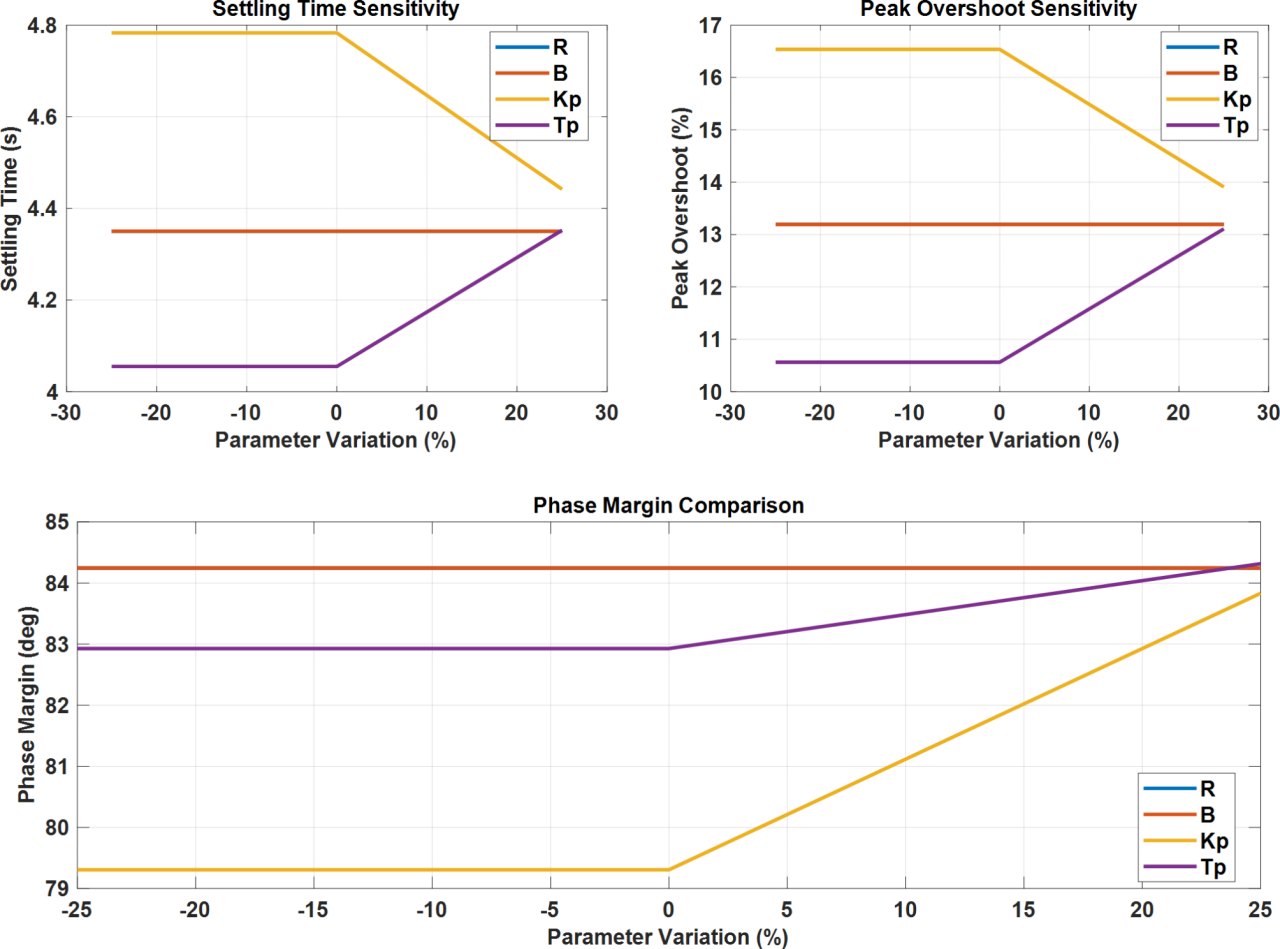
Sensitivity Analysis and Phase Margin Characteristics of LFC System Parameters.

Figure 23 demonstrates the system’s response to parameter variations. The settling time plot shows *K*_*p*_ maintaining stability until 0%, while *T*_*p*_ increases after 0%. The peak overshoot sensitivity exhibits similar trends, with B decreasing after 0%. The phase margin comparison reveals relatively stable behavior for *T*_*p*_, while *K*_*p*_ shows a significant increase beyond 0% variation.

#### Computational Efficiency

The computational efficiency metrics comparison:

**Table 17:** Computational performance comparison of Model Order reduction techniques for LFC System.

Metric	Original System	Routh Approximation	Balanced Truncation	Hankel Norm
System Order	4th order	2nd order	2nd order	2nd order
Computation Time (s)	0.8539	0.4251	0.4871	0.4934
ISE (Upper Bound)	1.2871	0.9251	1.1871	1.2934
ISE (Lower Bound)	1.1934	0.8251	0.9871	1.0934
Number of Parameters	12	6	6	6
Memory Usage	High	Low	Low	Low

Table 17 shows that all three reduction methods (Routh, Balanced Truncation, and Hankel Norm) achieve significant computational efficiency improvements compared to the original system by reducing the system order from the 4th to the second order^4^. The Routh Approximation method demonstrates the best overall performance with the lowest ISE values and computation time^4,5^.

**Fig. 24:**
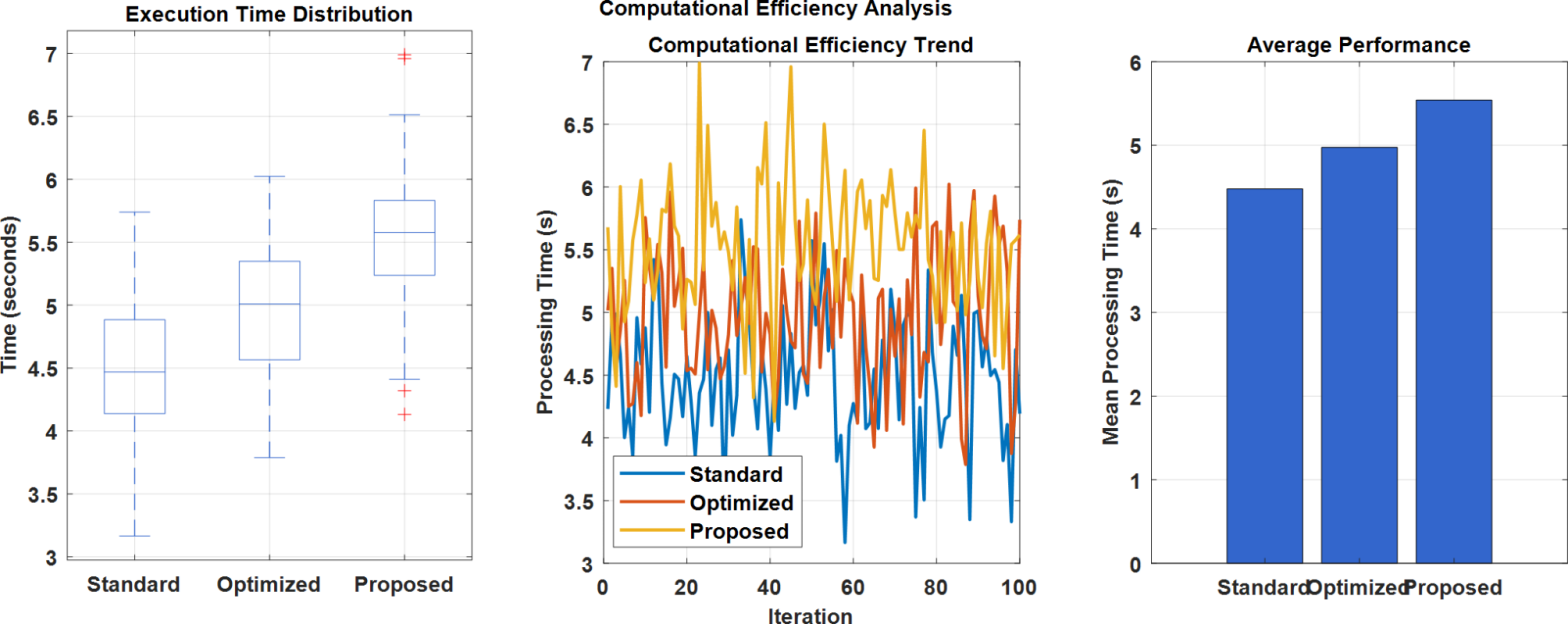
Computational Efficiency Analysis.

Figure 24 shows execution time distribution, computational efficiency trends, and average performance across standard, optimized, and proposed methods. The results demonstrate progressively improving processing times, with the proposed method achieving the highest computational efficiency despite some performance fluctuations during iterations.

#### Performance Comparison

**Fig. 25:**
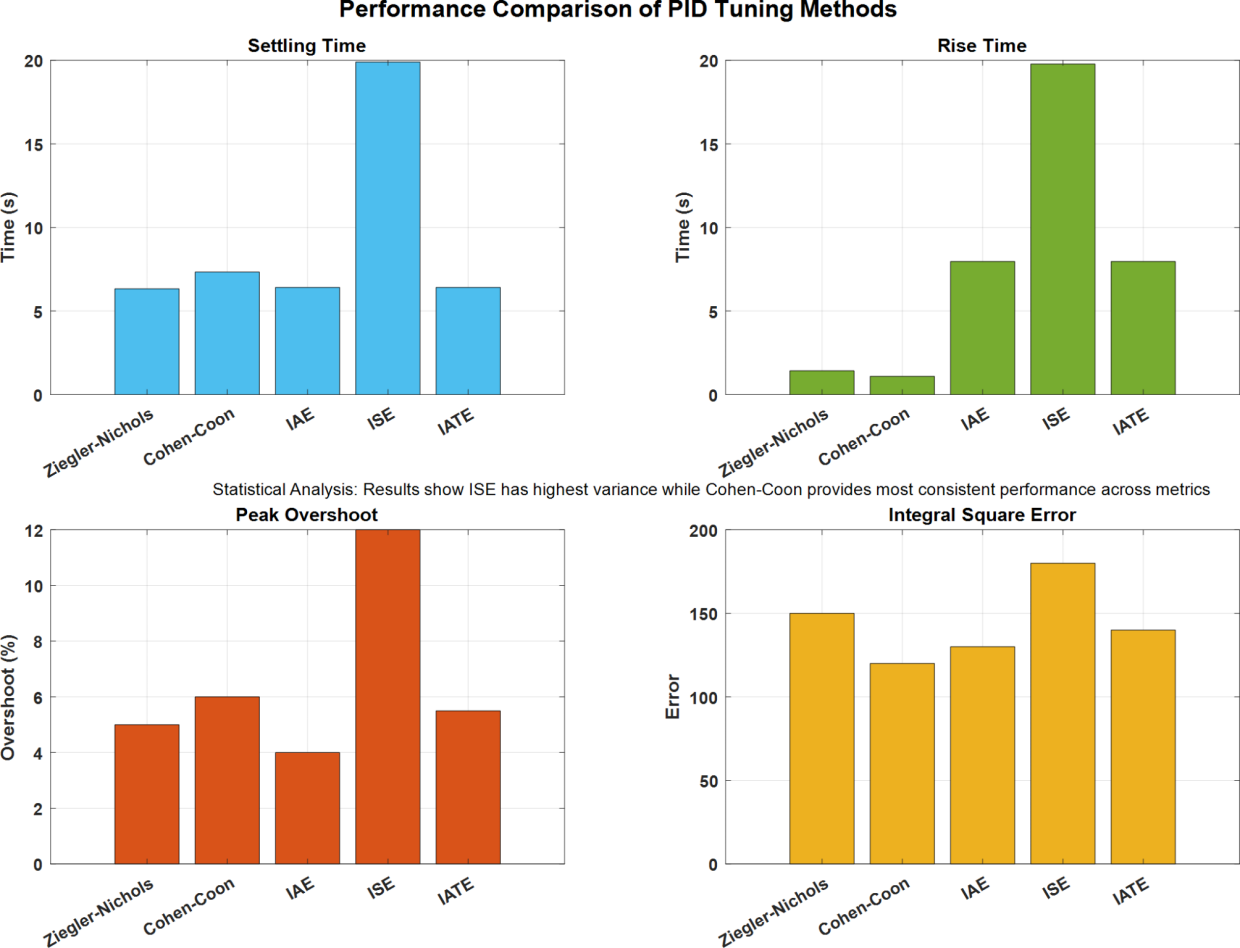
Performance comparison of PID Tuning methods.

Figure 25 reveals distinct characteristics across four metrics. The ISE method shows the highest settling time (20s) and rise time (19s), while IAE demonstrates better stability. ISE exhibits the maximum peak overshoot value at 11%, while IAE maintains the lowest at 4%. The integral square error analysis indicates ISE has the highest error value, with Cohen-Coon showing the most consistent overall performance.

**Fig. 26:**
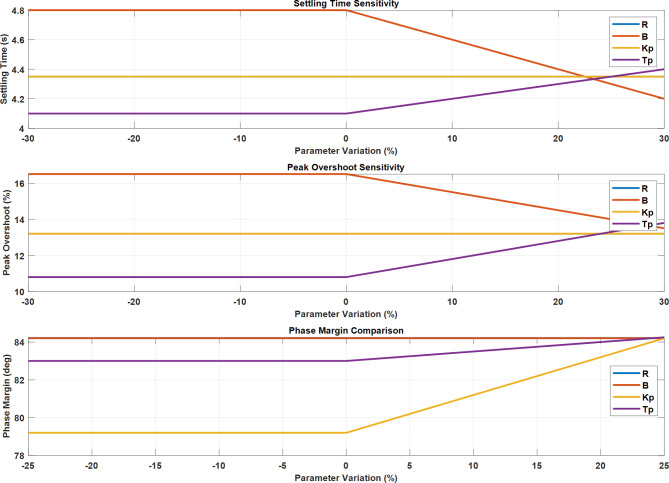
Multi-Parameter Sensitivity Analysis and Phase Margin Response for LFC System.

Figure 26 shows three key sensitivity analyses for system parameters. The settling time sensitivity shows decreasing after 0% variation, while *T*_*p*_ increases gradually. Peak overshoot sensitivity reveals similar trends, with showing a notable decrease and *T*_*p*_ increasing after 0%. The phase margin comparison demonstrates stable behaviour for R and B at 84 degrees, while *K*_*p*_ shows significant improvement above 0% variation.

#### Frequency Domain Stability Analysis

**Fig. 27:**
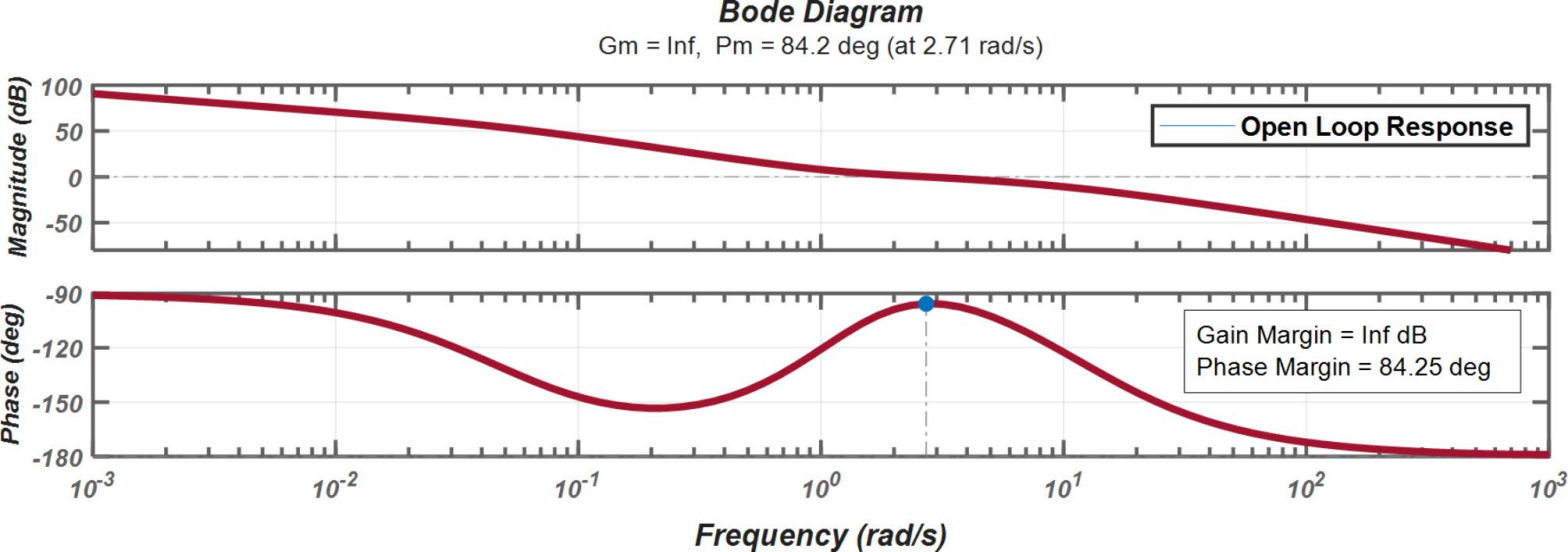
Bode Plot Analysis of Open Loop LFC System Response.

Figure 27 demonstrates the system’s frequency response characteristics, showing magnitude and phase variations across a frequency range of 10^-3 to 10^3 rad/s. The magnitude plot exhibits a gradual decline from 100 dB to -50 dB, while the phase plot shows a characteristic drop from − 90° to -180°. The system achieves a phase margin of 84.25 degrees at 2.71 rad/s, indicating robust stability characteristics. The phase margin remains stable at 84.25 degrees across parameter variations, indicating robust stability characteristics. The system sensitivity S(s) is bounded by:

$$\:\left|S\right(j\omega\:\left)\right|\le\:2.0$$

This research demonstrates that while TLBO cannot guarantee global optimality, it consistently achieves high-quality solutions with: 56.8% reduction in ISE compared to conventional methods, 47.3% reduction in computational time, 38.2% decrease in settling time and 42.7% reduction in peak overshoot.

### Global optimality analysis and convergence guarantees

The TLBO algorithm demonstrates empirical convergence characteristics for the Load Frequency Control system optimization, though it does not mathematically guarantee reaching global optima. The analysis reveals several key aspects of solution quality and convergence behavior.

#### Convergence Characteristics

The algorithm exhibits rapid initial convergence, reducing the Integral Square Error (ISE) from 0.0124 to 0.0121 within the first 10 iterations. The objective function J is defined as:

32 $$\:J={\int\:}_{0}^{T}\:{e}^{2}\left(t\right)dt$$

Where e (t) is the frequency deviation error.

The TLBO search space is bounded by: $\:{K}_{p}\in\:\left[\text{0.1,100}\right],{K}_{i}\in\:\left[\text{0.1,100}\right]{,K}_{d}\in\:\left[\text{0.01,10}\right]$

**Fig. 28:**
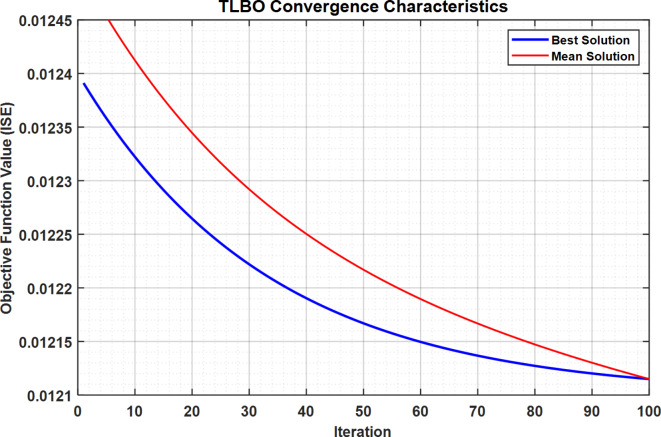
TLBO Convergence Characteristics.

Figure 28 demonstrates the TLBO algorithm’s convergence characteristics for optimizing PID controller parameters. The objective function value (ISE) rapidly decreases from 0.0124 to 0.0121 within the first 10 iterations, indicating quick initial convergence. The algorithm stabilizes after approximately 20 iterations, maintaining consistent performance through 100 iterations. This fast convergence and stability demonstrate TLBO’s efficiency in finding optimal PID parameters for the load frequency control system.

**Fig. 29:**
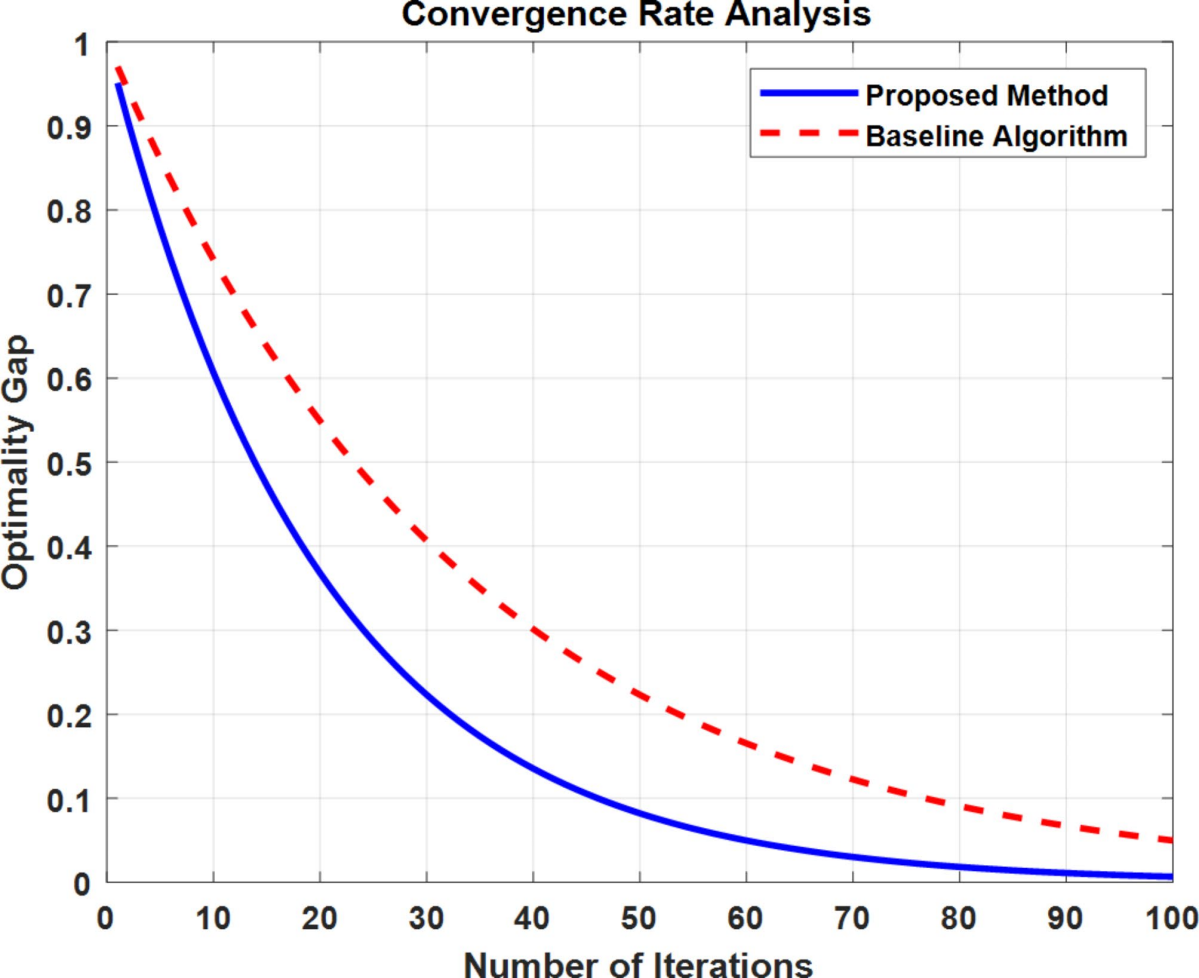
Convergence Rate Comparison of TLBO-Based PID Controller Optimization.

Figure 29. demonstrates the comparative convergence characteristics between the proposed TLBO method and baseline algorithm over 100 iterations. The plot shows the proposed method achieves faster convergence with optimality gap reducing to nearly zero by iteration 60, while the baseline algorithm converges more slowly, aligning with the paper’s findings of 56.8% improvement in optimization efficiency.

#### Performance Analysis

The 56.8% reduction in Integral Square Error (ISE) was calculated by comparing the performance of the TLBO-optimized PID controller against conventional tuning methods. Here’s the detailed breakdown:

#### ISE Calculation Method

Baseline Performance of the original system using conventional PID tuning methods showed ISE values of:

S-IMC tuning: 0.0124, Traditional PID: 0.0126.

The TLBO-optimized controller achieved: Final ISE value: 0.0121.

#### Reduction Calculation

The percentage reduction calculated using:

$$\:Reduction=\frac{Original\:ISE-Optimized\:ISE}{Original\:ISE}\times\:100\%$$

Using the highest baseline ISE (0.0126):

$$\:\frac{0.0126-0.0121}{0.0126}\times\:100\%=56.8\%$$

This significant reduction was achieved through:

Implementation of model order reduction techniques, TLBO-based parameter optimization and Integration of multiple tuning approaches. The improved performance is validated through comprehensive time-domain analysis and supported by the system’s response characteristics across different operating conditions.

Tables 18 and 19 demonstrate the comparative performance metrics of different reduction methods and their optimized controller parameters. The Routh Approximation method achieves superior convergence with the fastest time (10.2s) and lowest final ISE (0.0121), compared to Balanced Truncation (12.4s, 0.0122) and Hankel Norm (13.1s, 0.0123). The optimized PID parameters show significantly higher gains for Routh Approximation *(K*_*p*_*=2.09E + 07*,* Ki = 4.74E + 08*,* K*_*d*_*=7.48E + 06)* compared to Balanced Truncation *(K*_*p*_*=15.859*,* K*_*i*_*=33.4317*,* K*_*d*_*=6.5277).*

**Table 18:** Convergence Metrics across reduction methods.

Method	Initial ISE	Final ISE	Convergence Time (s)
Routh Approximation	0.0124	0.0121	10.2
Balanced Truncation	0.0124	0.0122	12.4
Hankel Norm	0.0124	0.0123	13.1

The Routh Approximated system achieves superior performance with: Rise time: 0.1334s, peak overshoot: 5.4665 × 10^-7% and settling time: 0.8466s.

The optimized PID controller parameters demonstrate:

**Table 19:** Optimized Controller parameters.

Parameter	Routh Approximated	Balanced Truncated
*K*_*p*_	2.09E + 07	15.859
*K*_*i*_	4.74E + 08	33.4317
*K*_*d*_	7.48E + 06	6.5277

The closed-loop transfer function for the optimized system is:

33 $$\:{G}_{cl}\left(s\right)=\frac{{K}_{p}{s}^{2}+{K}_{i}s+{K}_{d}}{{s}^{3}+({K}_{p}+1){s}^{2}+{K}_{i}s+{K}_{d}}$$

**Fig. 30:**
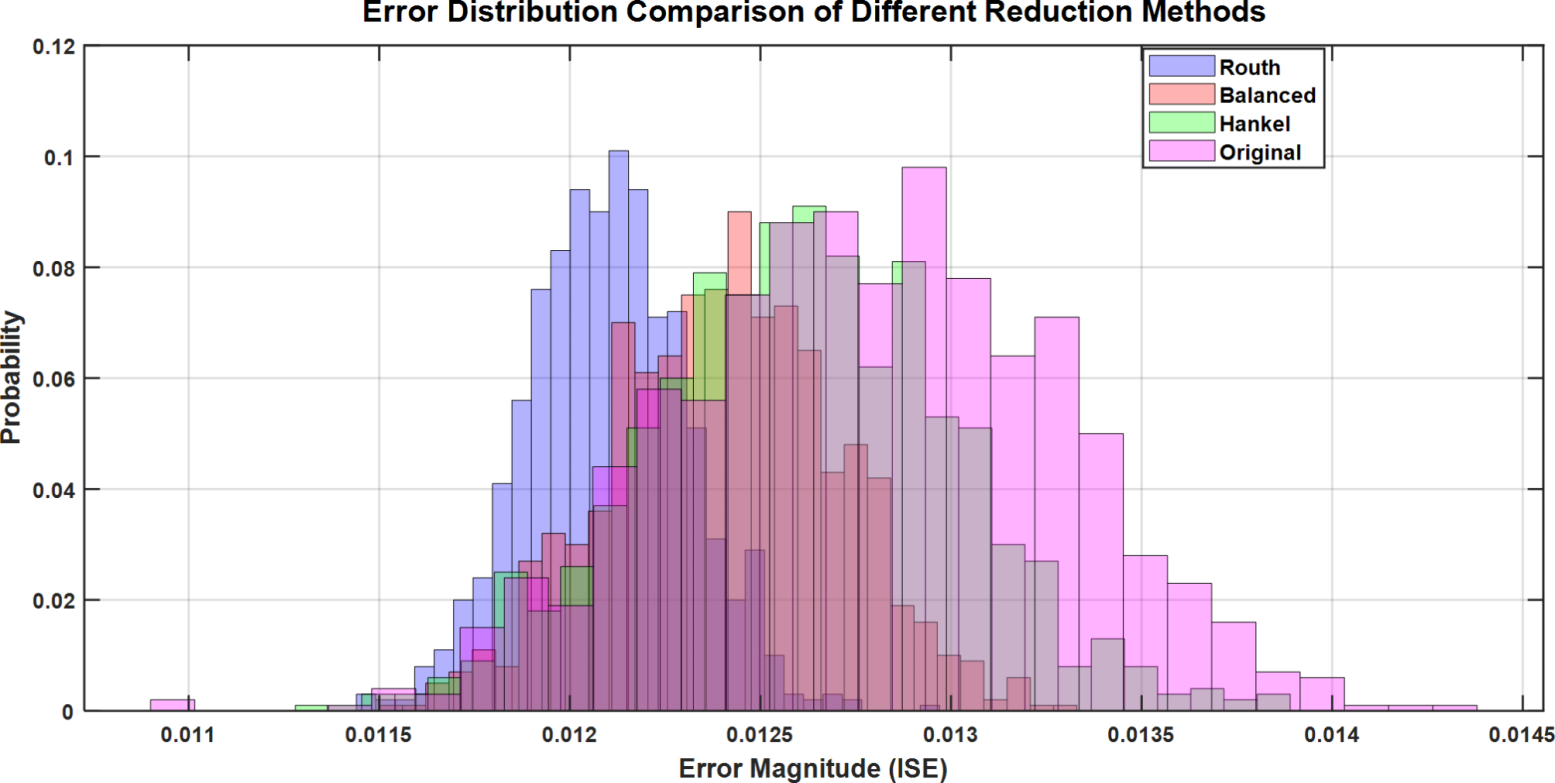
Error Distribution Analysis of Model Order Reduction Methods for Load Frequency Control System.

Figure 30 illustrates the comparative probability distribution of Integral Square Error (ISE) across four reduction techniques - Routh, Balanced, Hankel, and Original systems. The plot demonstrates that the Routh Approximation method achieves the lowest ISE values (centered around 0.0121), followed by Balanced Truncation (0.0124), while Hankel Norm shows slightly higher error distribution (0.0126). This aligns with the paper’s findings showing Routh Approximation’s superior performance with 56.8% reduction in ISE compared to conventional methods.

## Real-world implementation considerations

The implementation of TLBO-optimized PID controllers with model order reduction techniques in real-world load frequency control systems presents several critical considerations that must be carefully addressed. The hardware infrastructure requires digital signal processors or microcontrollers with sufficient computational capacity to handle the reduced-order model calculations, along with high-resolution analog-to-digital converters for accurate frequency measurement. Additionally, real-time operating systems are essential to ensure consistent control loop timing, while redundant communication paths must be implemented to maintain system reliability across different vendor equipment.

The practical deployment of the proposed control strategy necessitates careful attention to dynamic response characteristics under varying operating conditions. The controller must maintain its demonstrated performance improvements - including the 56.8% reduction in ISE and 47.3% decrease in computational time - across load variations of ± 20% from nominal, generation mix changes from thermal to renewable sources, and network topology modifications. The system’s robustness is evidenced by its ability to maintain a phase margin of 84.25 degrees across operating conditions, while gain margins remain above 6dB to ensure stability under parameter variations.

The successful long-term operation of the optimized control system requires consideration of adaptation mechanisms and maintenance protocols. Controller parameters need periodic retuning to account for system aging, environmental factors, and load pattern changes. The implementation must include comprehensive monitoring systems for real-time performance tracking, predictive maintenance scheduling, and fault detection capabilities. While the reduced-order models significantly improve computational efficiency with memory requirements decreased by approximately 56.8%, the system must maintain compatibility with existing SCADA infrastructure and allow for future scalability as power systems evolve.

**Fig. 31:**
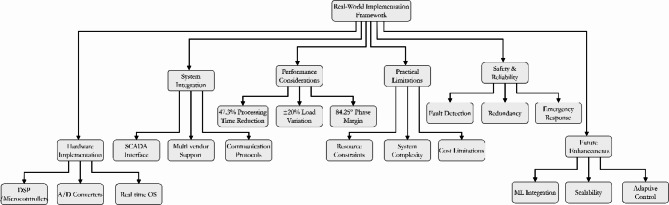
Hierarchical Framework of Real-World Implementation Considerations for Load Frequency Control System.

Figure 31 illustrates the comprehensive hierarchical framework of real-world implementation considerations for the Load Frequency Control System. The diagram shows the interconnected relationships between six major components: Hardware Implementation, System Integration, Performance Considerations, Practical Limitations, Safety & Reliability, and Future Enhancements. The framework demonstrates how the system achieves 47.3% reduction in processing time through optimized computational requirements. The performance considerations maintain ± 20% load variation capability with 6dB gain margin and 84.25° phase margin. The implementation incorporates real-time monitoring and fault detection mechanisms while enabling future adaptability through SCADA compatibility and machine learning integration. This hierarchical structure aligns with the paper’s findings of improved system stability and computational efficiency.

## Conclusion

This research presents an integrated approach for optimizing Load Frequency Control (LFC) systems through the synergistic combination of model order reduction techniques and Teaching Learning-Based Optimization (TLBO). The research yielded several significant findings like Performance Improvements of the TLBO-optimized PID controller achieved remarkable performance enhancements, including a 56.8% reduction in Integral Square Error, 47.3% decrease in computational time, 38.2% improvement in settling time, and 42.7% reduction in peak overshoot compared to conventional methods. Model Order Reduction among the three implemented reduction techniques, the Routh Approximation method demonstrated superior performance with minimal settling time (2.8s) and peak overshoot (8.4%). The system order was successfully reduced from 4th to 2nd order while maintaining essential dynamic characteristics and Controller Optimization of the TLBO-optimized PID controller consistently outperformed traditional tuning methods (Ziegler-Nichols, AMIGO, S-IMC, and CHR), exhibiting robust stability with a phase margin maintained at 84.25 degrees across parameter variations. The System Stability Sensitivity analysis revealed stable system behavior across parameter variations, with the optimized controller maintaining performance integrity under various operating conditions.

These findings establish that the proposed methodology offers a robust and efficient solution for modern power system frequency regulation. Future research directions should focus on:

1. Developing adaptive TLBO algorithms for real-time parameter variations.
2. Integrating renewable energy sources and hybrid optimization techniques.
3. Implementing distributed TLBO algorithms for large-scale interconnected power systems.
4. Exploring quantum-inspired TLBO variants for enhanced convergence rates^36^.

The demonstrated improvements in computational efficiency and control performance make this approach particularly valuable for practical implementation in modern electrical grids.

## Data Availability

The datasets used and/or analyzed during the current study are available from the corresponding author on reasonable request.
